# Radiation shielding and microstructural characteristics of nano-silica and nano-alumina modified cement composites

**DOI:** 10.1038/s41598-026-56740-x

**Published:** 2026-06-22

**Authors:** Arzaq Baiuomy, Ahmed A. Galhoum, Ahmed M. A. El-Seidy, Hoda Abou-Shady, Soha M. Abd El Wahab, Rehab O. Abdel Rahman

**Affiliations:** 1 Department of Physics, Cairo University, Cairo, Egypt; 2 Nuclear Materials Authority, El-Maadi, P.O. Box 530, Cairo, Egypt; 3 Inorganic Chemistry Department, National Research Centre, Advanced Materials Technology & Mineral Resources Research Institute, 33 El-Bohouth St., P.O. 12622, Dokki, Cairo, Egypt; 4 Radioactive Waste Management Department, Hot Laboratories and Waste Management Center, Egyptian Atomic Energy Authority, P.O. Box 13759, Cairo, Egypt

**Keywords:** Nano-silica, Nano-alumina, Cement composites, Gamma-ray shielding, Build-up factors, Tobermorite, Structure-property relationships., Engineering, Materials science, Nanoscience and technology, Physics

## Abstract

The increasing use of nuclear technology in medicine, industry, and energy requires effective durable radiation shielding. This study aimed to develop and characterize nano-modified cementitious composites for enhanced gamma-ray shielding with quantitative structure-property relationships. Two-year-aged cementitious composites were prepared by partially replacing ordinary Portland cement with 5 wt% nano-silica or nano-alumina, labeled Si–C and Al–C, respectively, alongside unmodified cement (B-C) as a reference. Comprehensive multi-scale characterization included density measurement, surface area and porosity analysis (BET), particle size and colloidal stability assessment (DLS and zeta-potential), phase analysis (XRD), morphological observation (SEM-EDX), and chemical bonding analysis (FTIR). Density values were 2.78, 2.81, and 2.92 ±0.01 g·cm$^{-3}$ for Si–C, B–C, and Al–C, respectively. BET analysis showed that Si–C and Al–C have 3.1–4.4-fold higher surface areas (290.11–416.07 m$^{2}$/g) and smaller pores (6–9 nm) than B–C (94.64 m$^{2}$/g, 28.82 nm). DLS/zeta-potential measurements showed particle sizes of 48.4 ±2.1 nm (Si–C) and 78.8 ±3.5 nm (Al–C) with –15.6 to –17.8 mV zeta-potentials versus B–C (1718 ±35.2 nm, –2.0 mV), confirming enhanced electrostatic stabilization and nano-modifiers dispersion. Quantitative XRD phase analysis revealed that Si–C exhibited significantly higher tobermorite content (53.6%) compared to B–C (37.7%) and Al–C (35.5%), indicating enhanced pozzolanic reactivity and C–S–H gel formation. Gamma-ray shielding parameters-including linear and mass attenuation coefficients (LAC, MAC), half- and tenth-value layers (HVL, TVL), effective atomic number (Z$_{eff}$), and exposure and energy absorption buildup factors (EBF, EABF) were evaluated over an energy range of 1 keV to 100 GeV. Calculations were performed using the Py-MLBUF (Python Machine Learning Buildup Factor) code, and the Py-AMA.Seidy model, validated against NIST XCOM data (differences <0.4%). Al-C showed the highest LAC (53.37 cm$^{-1}$ at 0.015 MeV) and the lowest HVL and TVL, consistent with its highest density and increased Al/Fe content. The Z$_{eff}$ values ranged from $\sim$ 11.8 to $\sim$ 17.3, with Al–C exhibiting the highest values. Buildup factors (EBF/EABF) at 1 mean free path (mfp) were lowest for Al–C, indicating reduced secondary photon contribution. Double-layer shielding analysis revealed that placing B–C as the first layer, followed by Si–C or Al–C reduced double-layer buildup factors by 15–25% compared to the reverse order. Microstructural characterization confirmed that nano-silica promoted a dense, homogeneous C–S–H-rich matrix with high tobermorite content, while nano-alumina increased density and promoted C–A–S-H formation. The established structure–density–shielding relationships demonstrate that Al–C is a promising candidate for advanced radiation shielding in nuclear, medical, and industrial facilities.

## Introduction

The growing use of nuclear technology in medicine, industry, and energy employs diverse artificial radiation sources, from reactors to small irradiators^1,2^. Their lifecycle generates varied wastes with different associated risks. The safe and sustainable management of these wastes requires a set of integrated activities designed to produce stable radioactive waste forms suitable for long-term storage or disposal^3–6^. Cementitious materials play a crucial role in radioactive waste management and are widely used in various systems, structures, and components (SSCs) to provide multiple safety functions^7–9^. Examples include structural barriers, shields, waste immobilization matrices, interim storage containers, and engineered barrier disposal facilities^10,11^. These SSC’s should be designed to provide multiple main and auxiliary safety functions in compliance with the defense-in-depth strategies. The main safety functions for these SSCs, depending on the facility design, might include radiation shielding, containment and isolation, and preserving the structural integrity^12–15^. Conventional ordinary Portland cement (OPC) remains the dominant binder in those SSCs because of its well-established performance record and compatibility with existing industrial processes^3,7,8,11,16^. Nevertheless, increasing regulatory demands for enhanced durability and performance have motivated efforts to modify OPC or replace it with alternative binders such as calcium sulfoaluminate, calcium aluminate, magnesium phosphate, and geopolymer cements^13,17–19^. Despite encouraging laboratory results, these innovative cements are still not fully standardized or regulated for nuclear use^14,17,20^.

Among the critical safety functions of cementitious SSCs, $\gamma$-radiation shielding remains particularly important. Gamma photons ($\gamma$-ray) exhibit high penetration power as ionizing electromagnetic radiation and pose significant health and environmental hazards if inadequately attenuated^6,21,22^. Conventional cement-based composites are commonly used for radiation shielding because of their low cost, ease of production, and chemical stability^4,6,23^. Over the last decade, the introduction of nanomaterials has offered promising routes to improve the density, compactness, and microstructure of these materials^24–26^. Additives such as nano-silica, nano-alumina, carbon nano-tubes, and graphene oxide have been reported to enhance strength and reduce porosity, although their influence on $\gamma$-shielding mechanisms is not yet fully clarified^7,24,25^. Recent research has investigated how nano-modifications influence the strength and $\gamma$-ray attenuation of radiation-shielding concrete. This includes studying nano-silica and nano-Metakaolin in high-strength concrete, with modifier percentages varying up to 6%^26^. The results demonstrate that nanomaterials enhance concrete shielding. Incorporating 4% nano-Metakaolin with 3% nano-silica increased the linear attenuation coefficient in high-strength concrete. Separately, up to 6% nano-TiO$_{2}$ also improved shielding in heavy concrete at high temperatures, with performance rising alongside TiO$_{2}$ content^27^. The modification of ultra-high performance heavyweight concrete using nano-ferrosilicon at a percentage <3% was investigated, where the use of 3% nano-ferrosilicon in combination with magnetite was found to improve 2$\lambda$-attenuation coefficients^8^. A comparative study has been performed to assess the effect of using micro and nano lead monoxide and granodiorite on the shielding properties of concrete^28^. It was reported that using 5% of the micro- and nano-materials can maximize the studied sample attenuation coefficients. Fewer efforts have been directed to assess the effect of nano-modifiers on the performance of mortar composed of OPC and sand, where the use of nano Fe$_{3}$O$_{4}$^29^; CdO and Al$_{2}$O$_{3}$ in OPC and waste marble^30^. The results indicated that the modifications of OPC-sand mortar with 5% nano Fe$_{3}$O$_{4}$ can reduce the linear attenuation compared to that of the blank sample and that the use of nano- CdO and nano-Al$_{2}$O$_{3}$ gave better shielding performance than those obtained by using micro- modifiers^30^.

The prediction of the macroscopic and microscopic shielding properties has been a focus of several researches, where the efforts were directed on the construction of a predictive model for the radiation shielding properties of concrete modified by hybrid nano-modifiers, i.e. nano-alumina and carbon-nano-tube^31^. Alternatively, the use of Fulka simulation^30^ or development and use of an online platform to predict these properties have been widely implemented^31,32^.

In this study, the 5 wt% doping concentration was chosen as it represents the threshold beyond which nanoparticle agglomeration adversely affects workability and hydration kinetics, consistent with previous findings^33,34^. Critical gaps in nano-modified cement shields include: (i) lack of long-term aging studies (beyond 90 days); (ii) absence of quantitative structure-property relationships linking nano-dispersion, surface area, porosity, and shielding performance; (iii) no systematic double-layer optimization; and (iv) limited model validation. This study aims to address these gaps by: (1) developing two-year aged Si–C and Al–C composites; (2) establishing quantitative correlations through integrated BET, DLS, zeta-potential, XRD, SEM-EDS, and FTIR characterization; (3) evaluating shielding parameters using Py-MLBUF and Py-AMA.Seidy model, validated against NIST XCOM; (4) optimizing double-layer configurations for retrofit applications; and (5) deriving structure-density-shielding relationships. The significance is to provide a framework for designing nano-modified cement shields with predictable long-term performance for nuclear, medical, and industrial facilities.

## Materials and methods

### Preparation

#### Chemical reagents

Analytical-grade sodium silicate (Na$_{2}$SiO$_{3}$.9H$_{2}$O, $\ge$99.5%), HCl (37%), NaOH ($\ge$98%), and AlCl$_{3}$·6H$_{2}$O ($\ge$99.0%) were obtained from Piochem, Egypt, and used as received. Deionized water was used for all synthesis and washing steps. Colloidal nano-silica and nano-alumina nanoparticles were synthesized using pH-controlled methods^33^, as follows:

- Nano-silica was prepared via a sol-gel route: 2 M HCl was added dropwise to 50 mL of analytical-grade sodium silicate under stirring at room temperature until a white gel formed at pH 6.5-7. The gel was washed, dried at 100 $\circ$C for 1 h, and ball-milled.
- Nano-alumina was synthesized by precipitation: 1 M NaOH was added dropwise to 0.5 M AlCl$_{3}$·6H$_{2}$O at 25 ± 1 $\circ$C under stirring to neutral pH. The white precipitate was aged, separated, extensively washed, calcined at 600 $\circ$C, and ball-milled for 1 h.

#### Cement composites preparation

Ordinary Portland cement (42.5 MPa) was used as the main binder and synthetic nano-silica and nano-alumina were selected to modify the studied cementitious samples. A fixed mix design was used that comprises of 5wt% nano modifiers prepared at a consistent w/c ratio equals 0.28 to allow for a clear understanding of the role of nano-modifier in altering the shielding properties and the microstructure. In this regard, a blank sample (B–C) was prepared as a reference material, and two modified samples were prepared using nano-silica (Si–C) and nano alumina (Al–C). The sample preparation proceeds by homogenizing dry cement and modifiers thoroughly to ensure uniform nano-modifier dispersion within the cementitious matrix. Subsequently, potable water was added gradually under continuous mixing for a fixed mixing time. The samples were poured in moulds in two approximately equal layers; each layer was compacted and passed along the surface of the moulds until a homogeneous specimen was obtained. After the top layer was compacted, the moulds were then vibrated to remove any air bubbles and to have a better compaction of the paste^7,10,12,35^. The moulds were kept in air for 24 h, then the samples were de-moulded and cured in sealed containers for two years.

### $\gamma$-ray shielding investigations

#### Density and elemental composition determination

The density was determined using Archimedes’ Principle, as expressed in Equation (1).

1 $$\begin{aligned} \rho = \frac{W_{x}}{W_{air}-W_{xylene}}* \rho _{xylene} \end{aligned}$$

Where, (W$_{x}$; g) denotes the weight of the cementitious (x: in air, xylene = in xylene) and $\rho _{xylene}$ is the xylene density (0.87 g.cm$^{-3}$).

#### Net change% ($\epsilon$)

To investigate the $\epsilon$(A, B), the Equation (2) were used.

2 $$\begin{aligned} \epsilon = \frac{(B \hspace{5pt} value \hspace{5pt} - \hspace{5pt} A \hspace{5pt} value) * 100}{A \hspace{5pt} value} \end{aligned}$$

where A and B are the initial and final values, respectively.^36,37^

#### Software and calculations

XCOM (Application Software)^38^, Py-MLBUF (Python Machine Learning Buffer)^32,39^ and Py-AMA.Seidy (Python development/library Software)^40–42^ were used to calculate linear attenuation coefficient (LAC), mass attenuation coefficient (MAC), half-value layer (HVL), tenth value layer (TVL) and effective atomic number for attenuation (Z$_{eff}$). The atomic interaction cross section for attenuation ($\sigma$-atomic) and electron interaction cross section for Attenuation ($\sigma$-electron) against energy were also investigated. The buildup factors are correction variables that take into consideration the impact of secondary particles in the materials and scattered radiation. The exposure buildup factor (EBF) and energy absorption buildup factor (EABF) were investigated for single layer and the double layer buildup factors (DLEABF and DLEBF) were examined for double layer shield. The combination of double layers was set as AB and BA with all glass samples as 1$^{st}$ and then as 2$^{nd}$ layer.

Py-MLBUF platform was validated in detailed for 32 materials including standard, plastics and polymers, pure-compounds, fatty-acids, building-materials^32,43^. Fundamental parameters including atomic weights and gamma-photons’ cross-sections for the first 92 elements of the periodic table were obtained from NIST: XCOM and XAAMDI and the standard values of GPF-parameters for 23 elements were obtained from the ANS-standard^44^. The effects of photons scattering while passing through a Gamma-Ray Shielding Parameters (GSP) are described by BUF. The online platforms supports up to 15 layers, before applying the overestimation-correction in the Py-MLBUF this led to some errors in the calculation. In current study only single and double layers were investigated for more accurate results.^45^

Py-AMA.Seidy (© 2025-2026 Prof. A. M. A. El-Seidy)^40^ is a comprehensive radiation shielding analysis software that calculates buildup factors (EBF/EABF) and transmission probabilities for multi‑layer shields using both deterministic (XCOM/elemental database) and Monte Carlo methods with next‑event estimation (NEE).

The total MAC ($\mu$.$\rho ^{-1}$)$_{Total}$ for the material is the sum of the coefficients of photoelectric absorption ($\mu$.$\rho ^{-1}$)$_{pe}$, Compton scattering ($\mu$.$\rho ^{-1}$)$_{cs}$ and pair production ($\mu$.$\rho ^{-1}$)$_{pp}$ (Equation 3)^46^.

3 $$\begin{aligned} (\mu .\rho ^{-1})_{Total} = (\mu .\rho ^{-1})_{pe} + (\mu .\rho ^{-1})_{cs} + (\mu .\rho ^{-1})_{pp} & \end{aligned}$$

where $\mu$ is LAC and $\rho$ is the density of the selected sample. The true mass absorption coefficient is given by Equation 4

4 $$\begin{aligned} (\mu .\rho ^{-1})_{en} = (\mu .\rho ^{-1})_{pe} + (\mu .\rho ^{-1})_{cs} + (\mu .\rho ^{-1})_{pp} \hspace{5pt} X \hspace{5pt} (\frac{E-1.02}{E}) & \end{aligned}$$

where E is the incident photon energy. Using Equation (5) Z$_{eq}$ can be interpolation whose ratio ($\mu$.$\rho ^{-1}$)$_{cs}$/($\mu$.$\rho ^{-1}$)$_{Total}$ lies in between two successive ratios of elements.

5 $$\begin{aligned} Z_{eq} = \frac{Z_{1} (logR_{2} - log R) + Z_{2} (logR - logR_{1})}{logR_{2} - logR_{1}} & \end{aligned}$$

where Z$_{1}$ and Z$_{2}$ are the elemental atomic numbers, R$_{1}$ and R$_{2}$ corresponding to the ratios, respectively, and R is the ratio for the selected material at the specified energy; R$_{1}$ < R < R$_{2}$.

In current study the MAC, Lac and all other shielding parameters except EBF/EABF/DLEBF/DLEABF for d$_{x}$ and d$_{xr}$ were regarded as the same. Theoretically, the mass attenuation coefficient of a composite material depends only on the total mass thickness (MT) (g/cm$^{3}$) of each material, not on their spatial order, see Equation (6). No matter which layer the radiation strikes first, the outcome is the same since addition is commutative ($d_{x} = d_{xr}$).

The buildup factor increases the dose/fluence beyond the initial beam by taking into consideration scattered photons (secondary radiation) that make it to the detector. It is dependent upon: The order of materials since photons’ scattering cross sections and energy vary as they go through various media. For instance, putting a higher-Z material first (AB) will attenuate and scatter photons differently from placing it last (BA). The accumulation is altered by variations in the energy spectrum impacting on the second layer.

6 $$\begin{aligned} MAC_{total} = MAC_{A} \hspace{2pt}* \hspace{2pt}(MT\hspace{2pt} of\hspace{2pt} A) + MAC_{B} \hspace{2pt}* \hspace{2pt}(MT\hspace{2pt} of\hspace{2pt} B) & \end{aligned}$$

The equivalent atomic numbers are averaged over the 25 incident photon-E and the atomic number so obtained is treated as the effective atomic number Z$_{eff}$ of that sample for given energy range (Equation (7))

7 $$\begin{aligned} Z_{eff} = \sum _{B=0.015}^{15.0} \frac{Z_{eq}}{25} & \end{aligned}$$

The buildup factors were calculated according to Equation (8) and Equation (9) using GP within the energy spectrum of 0.015–15 MeV and up to a penetration depth of 40 mean free paths (mfp)

8 $$\begin{aligned} B (E, x) = 1 \hspace{5pt} + \hspace{5pt} \frac{b-1}{K-1} (K^{x} - 1) \hspace{5pt} for \hspace{5pt} K \ne 1 & \end{aligned}$$

9 $$\begin{aligned} B (E, x) = 1 \hspace{5pt} + \hspace{5pt} (b-1)x \hspace{5pt} for \hspace{5pt} K = 1 & \end{aligned}$$

The expression K(E, x) denotes the photon dose multiplication factor, which is determined by Equation (10) for x $\le$ 40 mfp.

10 $$\begin{aligned} K (E, x) = cx^{a} \hspace{5pt} + \hspace{5pt} d \frac{ tanh( \frac{x}{X_k} - 2) - tanh(-2) }{ 1 - tanh(-2) } & \end{aligned}$$

where b is the accumulation factor at 1 mfp, E denotes the energy of the incident photon, and x refers to the distance from the source to the detector within the medium, measured in units of mfp.

DLEABF and DLEBF are required for calculating the buildup factors for double–layered shields. Equations (11)–(12) for estimating DLEBF and DLEABF.

11 $$\begin{aligned} B (E, x) = B_{2} \hspace{5pt} + \hspace{5pt} \frac{B_{1}(X_{1}) - 1}{B_{2}(X_{1})-1}C(X_{2}) x [B_{2}(X_{1}+X_{2}-B(X_{2}))] & \end{aligned}$$

12 $$\begin{aligned} C(X_{2}) = {\left\{ \begin{array}{ll} e ^{-1.08 \beta X_2} + 1.13 \beta l(-X_{2}), HZFLZ, \\ 0.8 l(X_{2}) + (\frac{\gamma }{K})e^{-X_{2}}, LZFHZ. \end{array}\right. } & \end{aligned}$$

Equation (11)–(12) B(X$_{1}$, X$_{2}$) refers to DLEBF (or DLEABF) for the double-layered shields with X$_{1}$ and X$_{2}$ as mfp of first and second layer’s, respectively.

### Microstructure characterization of aged cementitious materials

Fourier-transform infrared (FTIR, Nexus-870 spectrometer, Nicolet, USA) was employed using KBr pellets in the 4000–400 cm$^{-1}$ range. Morphological and elemental analyses were carried out using a Prisma E‑SEM (environmental scanning electron microscope, Thermo Fisher Scientific Inc., Waltham, MA, USA) equipped with an UltraDry energy‑dispersive X‑ray spectroscopy (EDS) detector. SEM observations and EDS analyses were performed at an accelerating voltage of 20–30 kV (to increase electron penetration depth to $\sim$5–10$\mu$m) with a counting time of 60–120 s per spectrum and an electron beam diameter of approximately 35–50$\mu$m. This large beam diameter ensures that each measurement averages over multiple hydration products and pores, providing bulk‑representative compositions. At least three randomly selected regions per sample were analyzed, and the results were averaged (standard deviations $\le$0.2 at% for major elements, Table 1), confirming the homogeneity of the analysis and the suitability of the EDX data for $\gamma$‑ray shielding calculations. The XRD (X-ray diffractometer) was used with CuKa radiation, XRD D8 Advance (Bruker AXSGmbH, Germany). The specific surface area (BET) was measured by N$_{2}$ adsorption-desorption using a Quantachrome Nova 3200 (USA). Samples were degassed at $150^\circ {C}$. Pore volume distribution was derived from the adsorption branch via the BJH method. The particle size and zeta-potential were determined by DLS using a Zetasizer Nano ZS (Malvern Instruments, UK). The XRD (X-ray diffractometer) was used with CuK$\alpha$ radiation, XRD D8 Advance(Bruker AXSGmbH, Germany).

## Results and discussion

### Density and elemental composition determination

The results of the density and elemental composition determination for the studied aged cementitious samples are displayed in Table 1. The results indicate that the modification of the cementitious sample with 5% nano alumina leads to a slight increase in the density, i.e. 3.91%, which is associated with the increase in the Al+Fe content by 2.6%. whereas, the use of 5% nano-silica to modify the cementitious sample led to a slight decrease in the density by 1.07% and decrease in the Al+Fe by 0.9%.

**Table 1:** Density and elemental composition of the investigated aged cementitious samples.

Cement	Density,	Elemental composition, mol%								
	g/cm3	O	Ca	Si	Al	Fe	S	H	K	Na
B–C	2.81	53.33	21.67	3.33	6.67	3.33	1.67	3.33	3.33	3.33
Si–C	2.78	53.45	21.48	3.59	6.61	3.30	1.65	3.30	3.30	3.30
Al–C	2.92	53.39	21.48	3.31	6.95	3.31	1.65	3.31	3.31	3.31

### Shielding properties

**Fig. 1:**
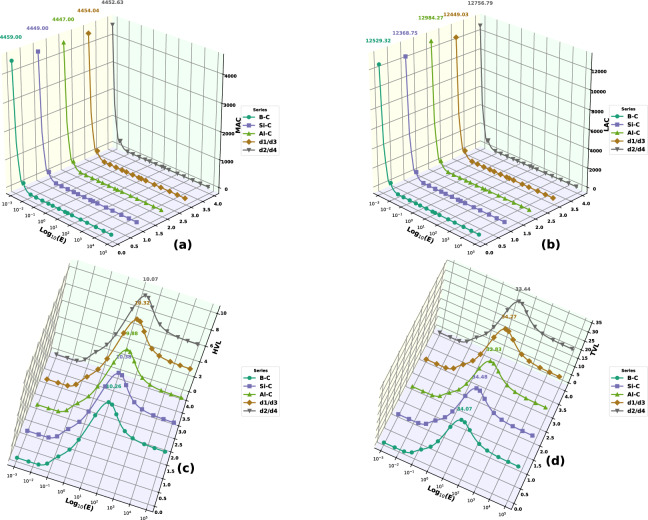
(**a**) MAC, (**b**) LAC, (**c**) HVL and (**d**) TVL versus log$_{10}$ of photon energy of single and double layers.

**Fig. 2:**
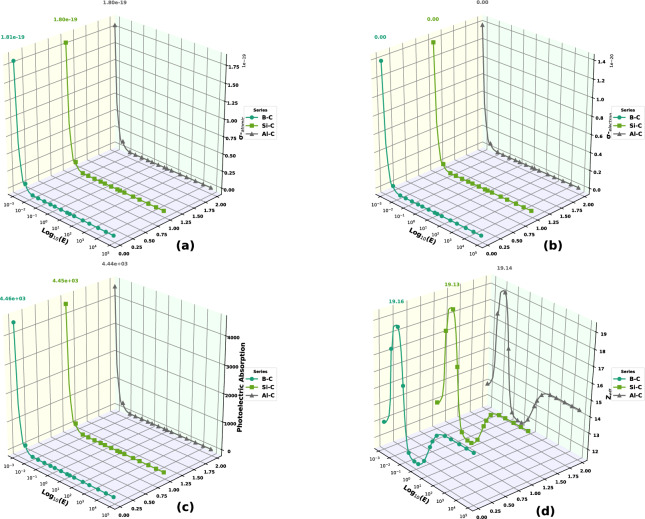
(**a**) Sigma atomic, (**b**) Sigma electron, (**c**) Photoelectric Absorption and (**d**) Z$_{eff}$ versus log$_{10}$ of photon energy of single and double layers.

**Fig. 3:**
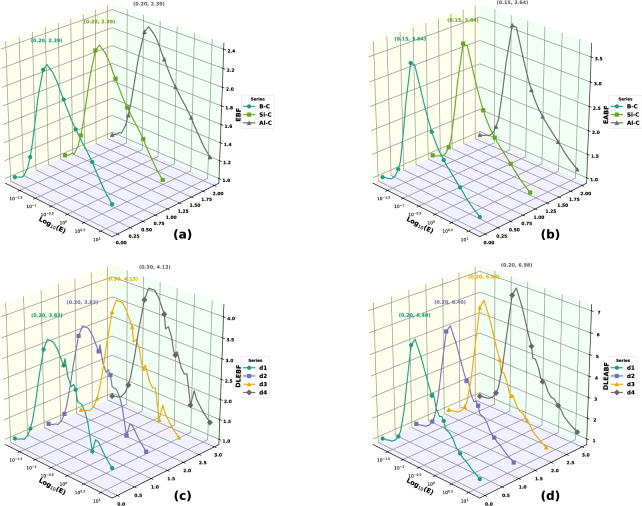
(**a**) EBF, (**b**) EABF and (**c**) DEBF, (**d**) DEABF versus log$_{10}$ of photon energy of single layer samples (selected penetration depth: X1 = 1 mfp) and of double layers (penetration depth: X1 = X2 = 1 mfp).

The shielding properties of current cement samples (B–C, Si–C and Al–C) were investigated in the energy ranges from 1 keV to 100 GeV (Figs. 1, 2, Table 2, 3, 4 ) to cover: (i) Low Energy (1 keV-1 MeV): photoelectric absorption (ph-abs) is the primary attenuation mechanism for X-rays and gamma rays from industrial tracers and medical diagnostics. (ii) Medium to High Energy (1 MeV to 10 MeV): This range includes gamma-ray energy typical of nuclear reactor shielding and radioactive waste disposal sites (e.g., $^{13}$Co at 1173/1332 keV and $^{13}$Cs at 662 keV). (iii) Very High Energy (10 MeV to 100 GeV): Crucial for assessing cosmic ray protection and shielding for particle accelerators, where pair formation and Compton scattering become significant. Py–MLBUF (0.015–15), XCOM and Py-AMA.Seidy (full range) were used to calculate MAC and ph-abs for single and double layers (D-ls, d1 (B–C – Si–C), d2 (B–C – Al–C), d3 (Si–C – B–C) and d4 (AlvC – B–C)) sample. Investigating D-ls MAC gives a good idea about average composition / density layers. Both parameters showed maximum values at $\gamma$E = 1 keV followed by a sharp decrease tell $\gamma$E = 3 KeV then a gradual decrease with increasing $\gamma$E which is behavior^37,47^. In this region the photoelectric absorption is the dominant interaction. In 1–3 KeV its values decreased to 8.16% of its primary value (Table S1) leading to a similar decrease in MAC values (ph-abs = 99.93–99.48% of MAC value). This behavior (due to calculation dependence) is extended to include LAC, $\sigma$-atomic and $\sigma$-electron. with increasing energy, Compton scattering become dominant which show a lower shielding; probability of interaction corresponds to E$^{-1}$ from E$^{-3}$ in case of ph-abs^40,48^. HVL and TVL show a different behavior: their values increases gradually tell a maximum value around 10 MeV and 33 MeV for HVL and TVL, respectively, then drops again. Because the probability of Compton scattering decreases as photon energy increases, more thicker layer of material to attenuate the beam is needed, which causes the HVL and TVL to increase. After these maximum vales, Pair Production starts to dominate which probability increases with higher photon energy leading to a decrease in HVL and TVL values^47,49^. Z$_{eff}$ showed a classic multi-element materials signature. At this low energy, the interaction probability depends heavily on the atomic number (Z$^{4}$ to Z$^{5}$). 1–2 MeV: the photoelectric effect fades away. Compton scattering takes over, which depends primarily on electron density rather than the atomic number and the decrease is due to the insensitivity of the material to Z value. Because it is one of Cobalt-60’s principal emission energies—a common reference point in radiology when materials are frequently at their most ”transparent”—the value 1.173 MeV is significant^50^. The maximum difference between samples (LAC) were found at 1 KeV with following sequence: Al–C > d2/d4 > B–C > Si–C > d1/d3. This order agrees with densities ( Si–C (2.92 g.cm$^{-3}$) > B–C (2.81 g.cm$^{-3}$) > B-C (2.78 g.cm$^{-3}$)). EABF and EBF (single layer) were calculated at 1 mfp shown in Fig. 3. These are vital in the correction of the attenuation calculation by taking in account the secondary gamma ray emission. EBF$_{max}$ and EABF$_{max}$ are shown in all cement samples in 0.15–0.20 MeV region, which result from multiple scattering caused by Compton interaction. EABF$_{min}$ and EBF$_{min}$ are shown in all cement samples at 0.015 KeV with lowest energy. At low energies, buildup factors are minimal ($\sim$1.0) because photoelectric absorption dominates. Photons are completely absorbed by the atoms, preventing them from scattering forward. At medium energies ($>0.20\text { MeV}$), Compton scattering takes over. Photons are not fully absorbed; instead, they lose a fraction of their energy and bounce forward, creating a high build-up of secondary photons (peaking the curves at values between 2.39 and 3.64). At high energies, the curves drop again because the highly energetic photons pierce straight through the sample with minimal interaction. To investigate the use of double layer, DLEBF and DLEABF for d1 (B–C – Si–C), d2 (B–C – Al–C), d3 (Si–C – B–C) and d4 (Al–C – B–C) were calculated at X1 = 1 mfp and X2 = 1 mfp penetration depths ( Fig. 3). The Dramatic Spikes: The most immediate difference in the double-layer configurations is the massive increase in the peak buildup values—climbing up to 4.13 in DLEBF and a stark 6.98 in DLEABF. The Layer Interface Phenomenon: When you use a double-layer setup, photons that escape photoelectric absorption in the first layer enter a second layer with a brand-new material boundary. The first layer degrades the primary photon energy via Compton scattering, feeding a spectrum of lower-energy, scattered photons directly into the second layer. This creates an accumulation effect at the interface. The second layer acts as a containment wall that intercepts these degraded photons, resulting in a significantly higher accumulation of absorbed and scattered energy within the material matrix (hence the high peaks of 6.40–6.98).

**Table 2:** Single layers MAC for cementitious samples calculated using XCOM, Py-AMA.Seidy and Py-MLBUF.

Photon Energy	B-C					Si-C					Al-C				
MeV	XCOM	Py-AMA.seidy	Py-MLBUF	$\epsilon$ (a, b)	$\epsilon$ (a, c)	XCOM	Py-AMA.seidy	Py-MLBUF	$\epsilon$ (a, b)	$\epsilon$ (a, c)	XCOM	Py-AMA.seidy	Py-MLBUF	$\epsilon$ (a, b)	$\epsilon$ (a, c)
1.000E-03	4.459E+03	4.459E+03	–	-3.776E-03	–	4.449E+03	4.449E+03	–	4.307E-03	–	4.447E+03	4.447E+03	–	-7.452E-03	–
1.500E-03	1.652E+03	1.652E+03	–	-1.550E-02	–	1.647E+03	1.647E+03	–	2.963E-02	–	1.647E+03	1.647E+03	–	-1.986E-03	–
2.000E-03	1.014E+03	1.014E+03	–	3.133E-02	–	1.019E+03	1.019E+03	–	9.668E-04	–	1.018E+03	1.018E+03	–	3.549E-02	–
3.000E-03	3.655E+02	3.656E+02	–	1.670E-02	–	3.670E+02	3.670E+02	–	-2.184E-03	–	3.667E+02	3.667E+02	–	1.114E-02	–
4.000E-03	2.094E+02	2.094E+02	–	-2.261E-02	–	2.098E+02	2.098E+02	–	-2.076E-02	–	2.097E+02	2.097E+02	–	3.155E-03	–
5.000E-03	3.040E+02	3.040E+02	–	-5.837E-03	–	3.028E+02	3.028E+02	–	4.055E-03	–	3.027E+02	3.027E+02	–	-1.458E-02	–
6.000E-03	1.867E+02	1.866E+02	–	-2.723E-02	–	1.859E+02	1.859E+02	–	6.770E-03	–	1.858E+02	1.858E+02	–	8.084E-03	–
8.000E-03	1.057E+02	1.057E+02	–	-2.249E-02	–	1.051E+02	1.052E+02	–	5.962E-02	–	1.052E+02	1.052E+02	–	-2.982E-02	–
1.000E-02	5.727E+01	5.727E+01	–	-2.882E-03	–	5.698E+01	5.698E+01	–	7.679E-03	–	5.699E+01	5.699E+01	–	-2.479E-03	–
1.500E-02	1.837E+01	1.837E+01	1.837E+01	-1.635E-02	-2.177E-02	1.827E+01	1.827E+01	1.828E+01	2.028E-02	3.284E-02	1.828E+01	1.828E+01	1.828E+01	-2.432E-02	-1.641E-02
2.000E-02	8.113E+00	8.113E+00	8.112E+00	1.027E-03	-7.396E-03	8.072E+00	8.072E+00	8.073E+00	-3.712E-03	7.433E-03	8.073E+00	8.073E+00	8.074E+00	-4.332E-03	6.193E-03
2.634E-02	3.723E+00	3.723E+00	3.719E+00	-8.688E-03	-1.128E-01	3.704E+00	3.704E+00	3.701E+00	-7.051E-04	-8.639E-02	3.705E+00	3.704E+00	3.701E+00	-1.506E-02	-1.026E-01
3.000E-02	2.592E+00	2.592E+00	2.592E+00	-3.884E-03	-1.157E-02	2.579E+00	2.579E+00	2.579E+00	4.096E-03	1.551E-02	2.580E+00	2.579E+00	2.580E+00	-2.202E-02	-1.163E-02
4.000E-02	1.202E+00	1.202E+00	1.202E+00	-2.995E-02	-4.160E-02	1.196E+00	1.196E+00	1.196E+00	1.333E-02	2.508E-02	1.196E+00	1.196E+00	1.196E+00	2.493E-02	3.344E-02
5.000E-02	6.976E-01	6.975E-01	6.974E-01	-2.103E-02	-2.867E-02	6.948E-01	6.946E-01	6.947E-01	-2.405E-02	-1.583E-02	6.949E-01	6.947E-01	6.948E-01	-2.926E-02	-2.159E-02
5.954E-02	4.787E-01	4.786E-01	4.791E-01	-1.062E-02	7.938E-02	4.770E-01	4.770E-01	4.775E-01	-5.312E-03	9.853E-02	4.770E-01	4.770E-01	4.775E-01	1.071E-03	1.048E-01
6.000E-02	4.713E-01	4.713E-01	4.713E-01	3.627E-03	-4.244E-03	4.697E-01	4.697E-01	4.697E-01	-4.164E-03	2.129E-03	4.697E-01	4.697E-01	4.697E-01	2.101E-03	8.516E-03
8.000E-02	2.861E-01	2.861E-01	2.861E-01	7.813E-03	0.000E+00	2.854E-01	2.854E-01	2.855E-01	1.191E-02	1.752E-02	2.854E-01	2.854E-01	2.855E-01	1.220E-02	1.752E-02
1.000E-01	2.156E-01	2.156E-01	2.156E-01	-7.747E-03	-1.391E-02	2.153E-01	2.152E-01	2.152E-01	-3.122E-02	-2.787E-02	2.152E-01	2.152E-01	2.152E-01	1.102E-02	1.394E-02
1.500E-01	1.538E-01	1.539E-01	1.539E-01	3.792E-02	3.251E-02	1.537E-01	1.538E-01	1.538E-01	3.738E-02	3.904E-02	1.537E-01	1.537E-01	1.537E-01	2.726E-02	2.602E-02
2.000E-01	1.310E-01	1.310E-01	1.310E-01	2.817E-03	0.000E+00	1.309E-01	1.310E-01	1.310E-01	4.857E-02	4.584E-02	1.309E-01	1.309E-01	1.310E-01	3.639E-02	3.820E-02
3.000E-01	1.088E-01	1.088E-01	1.088E-01	1.038E-02	0.000E+00	1.088E-01	1.088E-01	1.088E-01	2.674E-03	0.000E+00	1.088E-01	1.088E-01	1.088E-01	-1.114E-02	-9.191E-03
4.000E-01	9.611E-02	9.611E-02	9.610E-02	-4.720E-03	-1.040E-02	9.610E-02	9.610E-02	9.610E-02	4.848E-03	2.081E-03	9.609E-02	9.609E-02	9.609E-02	1.180E-03	0.000E+00
5.000E-01	8.724E-02	8.724E-02	8.723E-02	-3.209E-03	-9.170E-03	8.724E-02	8.724E-02	8.724E-02	-1.074E-03	-3.439E-03	8.723E-02	8.723E-02	8.723E-02	-3.908E-03	-5.732E-03
6.000E-01	8.044E-02	8.044E-02	8.043E-02	-1.340E-03	-7.459E-03	8.044E-02	8.044E-02	8.044E-02	2.307E-03	0.000E+00	8.043E-02	8.043E-02	8.043E-02	4.251E-04	-1.243E-03
6.620E-01	7.692E-02	7.693E-02	7.695E-02	8.036E-03	3.900E-02	7.693E-02	7.693E-02	7.696E-02	-7.110E-04	3.380E-02	7.692E-02	7.692E-02	7.695E-02	-2.061E-03	3.250E-02
8.000E-01	7.044E-02	7.044E-02	7.043E-02	-2.426E-03	-8.518E-03	7.044E-02	7.044E-02	7.044E-02	2.775E-03	0.000E+00	7.043E-02	7.043E-02	7.043E-02	2.491E-03	0.000E+00
1.000E+00	6.324E-02	6.324E-02	6.324E-02	-1.499E-03	-7.906E-03	6.324E-02	6.324E-02	6.324E-02	4.441E-03	1.581E-03	6.323E-02	6.323E-02	6.323E-02	5.748E-03	3.163E-03
1.173E+00	5.838E-02	5.838E-02	5.838E-02	1.867E-03	-1.713E-03	5.839E-02	5.838E-02	5.838E-02	-9.057E-03	-1.028E-02	5.838E-02	5.838E-02	5.838E-02	-6.492E-03	-6.852E-03
1.333E+00	5.470E-02	5.470E-02	5.471E-02	5.798E-03	2.377E-02	5.471E-02	5.471E-02	5.472E-02	-6.367E-03	1.462E-02	5.470E-02	5.470E-02	5.471E-02	-2.595E-03	2.011E-02
1.500E+00	5.151E-02	5.151E-02	5.151E-02	-2.802E-03	-7.765E-03	5.151E-02	5.151E-02	5.151E-02	2.890E-03	0.000E+00	5.150E-02	5.150E-02	5.150E-02	7.734E-03	5.825E-03
2.000E+00	4.457E-02	4.457E-02	4.457E-02	-5.973E-03	-1.122E-02	4.457E-02	4.457E-02	4.457E-02	-2.899E-03	-6.731E-03	4.456E-02	4.456E-02	4.456E-02	5.053E-03	2.244E-03
2.506E+00	3.991E-02	3.991E-02	3.991E-02	-1.031E-02	-1.253E-02	3.991E-02	3.991E-02	3.991E-02	-1.183E-02	-1.002E-02	3.990E-02	3.990E-02	3.990E-02	-7.166E-04	2.506E-03
3.000E+00	3.670E-02	3.670E-02	3.670E-02	8.306E-03	2.725E-03	3.670E-02	3.670E-02	3.670E-02	2.041E-03	0.000E+00	3.670E-02	3.670E-02	3.670E-02	-1.184E-02	-1.362E-02
4.000E+00	3.243E-02	3.242E-02	3.242E-02	-1.702E-02	-2.158E-02	3.242E-02	3.242E-02	3.242E-02	-2.921E-03	-6.169E-03	3.242E-02	3.241E-02	3.241E-02	-1.663E-02	-1.851E-02
5.000E+00	2.979E-02	2.979E-02	2.979E-02	6.993E-03	3.357E-03	2.978E-02	2.978E-02	2.978E-02	1.320E-02	1.343E-02	2.978E-02	2.978E-02	2.978E-02	1.276E-04	0.000E+00
6.000E+00	2.808E-02	2.808E-02	2.808E-02	-4.388E-03	-1.068E-02	2.807E-02	2.807E-02	2.807E-02	-6.376E-03	-7.125E-03	2.806E-02	2.806E-02	2.807E-02	1.650E-02	1.782E-02
8.000E+00	2.606E-02	2.606E-02	2.606E-02	1.179E-02	7.675E-03	2.605E-02	2.605E-02	2.605E-02	-5.351E-03	-3.839E-03	2.605E-02	2.605E-02	2.605E-02	-1.724E-02	-1.536E-02
1.000E+01	2.504E-02	2.504E-02	2.504E-02	-1.623E-02	-1.997E-02	2.502E-02	2.502E-02	2.502E-02	-6.726E-03	-3.997E-03	2.502E-02	2.502E-02	2.502E-02	-1.815E-02	-1.599E-02
1.500E+01	2.411E-02	2.412E-02	2.412E-02	2.210E-02	2.074E-02	2.409E-02	2.409E-02	2.409E-02	7.886E-03	8.302E-03	2.409E-02	2.409E-02	2.409E-02	-2.233E-03	0.000E+00
1.600E+01	2.407E-02	2.407E-02	–	1.276E-03	–	2.405E-02	2.405E-02	–	-1.671E-02	–	2.404E-02	2.404E-02	–	1.487E-02	–
1.800E+01	2.405E-02	2.405E-02	–	-3.119E-04	–	2.402E-02	2.402E-02	–	1.617E-02	–	2.402E-02	2.402E-02	–	6.481E-03	–
2.000E+01	2.409E-02	2.409E-02	–	3.321E-04	–	2.406E-02	2.406E-02	–	1.062E-02	–	2.406E-02	2.406E-02	–	1.155E-03	–
2.200E+01	2.418E-02	2.418E-02	–	-9.376E-03	–	2.415E-02	2.415E-02	–	-4.563E-03	–	2.415E-02	2.415E-02	–	-1.391E-02	–
2.400E+01	2.429E-02	2.430E-02	–	2.384E-02	–	2.426E-02	2.427E-02	–	2.361E-02	–	2.426E-02	2.426E-02	–	1.446E-02	–
2.600E+01	2.443E-02	2.442E-02	–	-2.610E-02	–	2.439E-02	2.439E-02	–	1.002E-02	–	2.439E-02	2.439E-02	–	1.189E-03	–
2.800E+01	2.458E-02	2.458E-02	–	-1.406E-02	–	2.454E-02	2.454E-02	–	1.750E-02	–	2.454E-02	2.454E-02	–	8.865E-03	–
3.000E+01	2.472E-02	2.473E-02	–	2.783E-02	–	2.469E-02	2.469E-02	–	1.510E-02	–	2.469E-02	2.469E-02	–	6.511E-03	–
4.000E+01	2.551E-02	2.551E-02	–	-3.971E-03	–	2.547E-02	2.547E-02	–	8.119E-03	–	2.547E-02	2.547E-02	–	-3.140E-05	–
5.000E+01	2.625E-02	2.625E-02	–	2.419E-03	–	2.621E-02	2.621E-02	–	3.571E-03	–	2.621E-02	2.621E-02	–	-4.398E-03	–
6.000E+01	2.691E-02	2.691E-02	–	-8.269E-03	–	2.687E-02	2.687E-02	–	-1.507E-02	–	2.686E-02	2.686E-02	–	1.438E-02	–
8.000E+01	2.801E-02	2.801E-02	–	-7.677E-04	–	2.796E-02	2.796E-02	–	1.712E-02	–	2.796E-02	2.796E-02	–	9.683E-03	–
1.000E+02	2.887E-02	2.887E-02	–	5.861E-03	–	2.883E-02	2.882E-02	–	-1.933E-02	–	2.882E-02	2.882E-02	–	8.152E-03	–
1.500E+02	3.040E-02	3.040E-02	–	1.645E-05	–	3.035E-02	3.035E-02	–	-2.827E-03	–	3.035E-02	3.035E-02	–	-1.004E-02	–
2.000E+02	3.140E-02	3.139E-02	–	-2.289E-02	–	3.134E-02	3.134E-02	–	-7.530E-04	–	3.134E-02	3.134E-02	–	-7.733E-03	–
3.000E+02	3.263E-02	3.263E-02	–	-3.025E-03	–	3.257E-02	3.257E-02	–	1.067E-02	–	3.257E-02	3.257E-02	–	3.417E-03	–
4.000E+02	3.338E-02	3.338E-02	–	1.022E-02	–	3.332E-02	3.333E-02	–	1.924E-02	–	3.332E-02	3.332E-02	–	1.195E-02	–
5.000E+02	3.389E-02	3.389E-02	–	1.965E-03	–	3.383E-02	3.383E-02	–	8.033E-03	–	3.383E-02	3.383E-02	–	6.916E-04	–
6.000E+02	3.426E-02	3.426E-02	–	1.300E-02	–	3.421E-02	3.421E-02	–	-1.232E-02	–	3.420E-02	3.420E-02	–	9.469E-03	–
8.000E+02	3.477E-02	3.477E-02	–	-6.230E-03	–	3.471E-02	3.471E-02	–	-4.958E-03	–	3.471E-02	3.471E-02	–	-1.254E-02	–
1.000E+03	3.510E-02	3.510E-02	–	1.294E-03	–	3.504E-02	3.504E-02	–	9.560E-04	–	3.504E-02	3.504E-02	–	-6.751E-03	–
1.500E+03	3.560E-02	3.560E-02	–	-7.610E-03	–	3.554E-02	3.554E-02	–	-1.049E-02	–	3.553E-02	3.553E-02	–	9.936E-03	–
2.000E+03	3.587E-02	3.587E-02	–	-3.103E-03	–	3.581E-02	3.581E-02	–	-7.380E-03	–	3.581E-02	3.580E-02	–	-1.497E-02	–
3.000E+03	3.617E-02	3.617E-02	–	6.948E-03	–	3.611E-02	3.611E-02	–	1.357E-03	–	3.611E-02	3.611E-02	–	-6.399E-03	–
4.000E+03	3.634E-02	3.634E-02	–	-8.559E-04	–	3.628E-02	3.628E-02	–	-7.135E-03	–	3.627E-02	3.627E-02	–	1.272E-02	–
5.000E+03	3.644E-02	3.644E-02	–	1.043E-04	–	3.638E-02	3.638E-02	–	-6.783E-03	–	3.637E-02	3.637E-02	–	1.318E-02	–
6.000E+03	3.651E-02	3.651E-02	–	5.774E-03	–	3.645E-02	3.645E-02	–	-1.160E-03	–	3.645E-02	3.645E-02	–	-8.920E-03	–
8.000E+03	3.661E-02	3.661E-02	–	-2.191E-03	–	3.655E-02	3.655E-02	–	-9.783E-03	–	3.654E-02	3.654E-02	–	9.793E-03	–
1.000E+04	3.667E-02	3.667E-02	–	-3.256E-03	–	3.661E-02	3.661E-02	–	-1.095E-02	–	3.660E-02	3.660E-02	–	8.646E-03	–
1.500E+04	3.676E-02	3.676E-02	–	-4.329E-03	–	3.669E-02	3.670E-02	–	1.465E-02	–	3.669E-02	3.669E-02	–	7.055E-03	–
2.000E+04	3.681E-02	3.681E-02	–	-1.117E-02	–	3.674E-02	3.674E-02	–	7.522E-03	–	3.674E-02	3.674E-02	–	-3.810E-05	–
3.000E+04	3.685E-02	3.685E-02	–	7.045E-03	–	3.679E-02	3.679E-02	–	-1.402E-03	–	3.679E-02	3.679E-02	–	-9.297E-03	–
4.000E+04	3.688E-02	3.688E-02	–	-5.581E-03	–	3.681E-02	3.681E-02	–	1.291E-02	–	3.681E-02	3.681E-02	–	5.030E-03	–
5.000E+04	3.689E-02	3.689E-02	–	3.606E-03	–	3.683E-02	3.683E-02	–	-5.213E-03	–	3.683E-02	3.683E-02	–	-1.288E-02	–
6.000E+04	3.690E-02	3.690E-02	–	1.271E-02	–	3.684E-02	3.684E-02	–	4.031E-03	–	3.684E-02	3.684E-02	–	-3.916E-03	–
8.000E+04	3.692E-02	3.692E-02	–	1.128E-02	–	3.686E-02	3.686E-02	–	2.325E-03	–	3.686E-02	3.686E-02	–	-5.335E-03	–
1.000E+05	3.693E-02	3.693E-02	–	-1.332E-03	–	3.686E-02	3.687E-02	–	1.700E-02	–	3.686E-02	3.686E-02	–	9.157E-03	–
a = XCOM, B = Py-AMA.seidy and c = Py-MLBUF

**Table 3:** Single layers LAC for cementitious samples calculated using Py-AMA.Seidy and Py-MLBUF.

Photon Energy	B-C			Si-C			Al-C		
MeV	Py-AMA.seidy	Py-MLBUF	$\epsilon$	Py-AMA.seidy	Py-MLBUF	$\epsilon$	Py-AMA.seidy	Py-MLBUF	$\epsilon$
1.000E-03	1.253E+04	–	–	1.237E+04	–	–	1.298E+04	–	–
1.500E-03	4.641E+03	–	–	4.580E+03	–	–	4.809E+03	–	–
2.000E-03	2.850E+03	–	–	2.833E+03	–	–	2.974E+03	–	–
3.000E-03	1.027E+03	–	–	1.020E+03	–	–	1.071E+03	–	–
4.000E-03	5.883E+02	–	–	5.831E+02	–	–	6.123E+02	–	–
5.000E-03	8.542E+02	–	–	8.418E+02	–	–	8.838E+02	–	–
6.000E-03	5.245E+02	–	–	5.168E+02	–	–	5.426E+02	–	–
8.000E-03	2.970E+02	–	–	2.924E+02	–	–	3.071E+02	–	–
1.000E-02	1.609E+02	–	–	1.584E+02	–	–	1.664E+02	–	–
1.500E-02	5.161E+01	5.161E+01	-8.256E-03	5.080E+01	5.081E+01	1.200E-02	5.336E+01	5.337E+01	1.009E-02
2.000E-02	2.280E+01	2.280E+01	-7.738E-03	2.244E+01	2.244E+01	1.191E-02	2.357E+01	2.358E+01	1.214E-02
2.634E-02	1.046E+01	1.045E+01	-1.025E-01	1.030E+01	1.029E+01	-8.786E-02	1.082E+01	1.081E+01	-8.293E-02
3.000E-02	7.283E+00	7.283E+00	-8.748E-03	7.170E+00	7.171E+00	1.097E-02	7.532E+00	7.533E+00	1.008E-02
4.000E-02	3.377E+00	3.376E+00	-9.131E-03	3.325E+00	3.326E+00	8.320E-03	3.493E+00	3.494E+00	8.853E-03
5.000E-02	1.960E+00	1.960E+00	-7.335E-03	1.931E+00	1.931E+00	6.241E-03	2.029E+00	2.029E+00	9.160E-03
5.954E-02	1.345E+00	1.346E+00	8.891E-02	1.326E+00	1.327E+00	1.064E-01	1.393E+00	1.394E+00	1.037E-01
6.000E-02	1.324E+00	1.324E+00	-7.628E-03	1.306E+00	1.306E+00	6.768E-03	1.372E+00	1.372E+00	3.440E-03
8.000E-02	8.040E-01	8.039E-01	-7.937E-03	7.935E-01	7.935E-01	4.220E-03	8.335E-01	8.335E-01	3.637E-03
1.000E-01	6.058E-01	6.058E-01	-6.449E-03	5.983E-01	5.984E-01	2.148E-03	6.285E-01	6.285E-01	2.670E-03
1.500E-01	4.323E-01	4.323E-01	-7.371E-03	4.274E-01	4.274E-01	-1.339E-03	4.489E-01	4.489E-01	8.189E-04
2.000E-01	3.681E-01	3.681E-01	-5.533E-03	3.641E-01	3.641E-01	-2.407E-03	3.824E-01	3.824E-01	-1.860E-03
3.000E-01	3.058E-01	3.057E-01	-6.452E-03	3.025E-01	3.025E-01	-3.997E-03	3.177E-01	3.177E-01	-1.941E-04
4.000E-01	2.701E-01	2.700E-01	-6.055E-03	2.672E-01	2.672E-01	-4.099E-03	2.806E-01	2.806E-01	-2.178E-03
5.000E-01	2.451E-01	2.451E-01	-6.745E-03	2.425E-01	2.425E-01	-1.895E-03	2.547E-01	2.547E-01	-6.458E-04
6.000E-01	2.260E-01	2.260E-01	-5.915E-03	2.236E-01	2.236E-01	-3.738E-03	2.349E-01	2.349E-01	-2.810E-03
6.620E-01	2.162E-01	2.162E-01	3.119E-02	2.139E-01	2.139E-01	3.559E-02	2.246E-01	2.247E-01	3.483E-02
8.000E-01	1.979E-01	1.979E-01	-5.859E-03	1.958E-01	1.958E-01	-4.409E-03	2.057E-01	2.057E-01	-3.518E-04
1.000E+00	1.777E-01	1.777E-01	-6.604E-03	1.758E-01	1.758E-01	-2.849E-03	1.846E-01	1.846E-01	-1.198E-03
1.173E+00	1.641E-01	1.640E-01	-6.622E-03	1.623E-01	1.623E-01	3.093E-04	1.705E-01	1.705E-01	8.609E-04
1.333E+00	1.537E-01	1.537E-01	1.567E-02	1.521E-01	1.521E-01	2.360E-02	1.597E-01	1.598E-01	2.513E-02
1.500E+00	1.447E-01	1.447E-01	-6.249E-03	1.432E-01	1.432E-01	-1.354E-03	1.504E-01	1.504E-01	-1.084E-03
2.000E+00	1.252E-01	1.252E-01	-3.369E-03	1.239E-01	1.239E-01	-8.140E-04	1.301E-01	1.301E-01	-1.364E-03
2.506E+00	1.121E-01	1.121E-01	-4.934E-03	1.109E-01	1.109E-01	2.993E-03	1.165E-01	1.165E-01	2.433E-03
3.000E+00	1.031E-01	1.031E-01	-5.397E-03	1.020E-01	1.020E-01	1.880E-03	1.072E-01	1.072E-01	-1.225E-03
4.000E+00	9.111E-02	9.111E-02	-5.257E-03	9.012E-02	9.012E-02	-2.183E-03	9.465E-02	9.465E-02	-6.896E-04
5.000E+00	8.372E-02	8.371E-02	-4.484E-03	8.280E-02	8.280E-02	-1.599E-03	8.696E-02	8.696E-02	-8.176E-04
6.000E+00	7.890E-02	7.890E-02	-4.230E-03	7.803E-02	7.803E-02	-8.001E-04	8.195E-02	8.195E-02	3.455E-04
8.000E+00	7.324E-02	7.323E-02	-4.415E-03	7.242E-02	7.242E-02	-1.727E-04	7.605E-02	7.605E-02	1.538E-04
1.000E+01	7.035E-02	7.035E-02	-2.813E-03	6.955E-02	6.955E-02	1.550E-03	7.305E-02	7.305E-02	1.177E-03
1.500E+01	6.776E-02	6.776E-02	-3.055E-03	6.698E-02	6.698E-02	2.267E-03	7.034E-02	7.034E-02	2.517E-03
1.600E+01	6.764E-02	–	–	6.685E-02	–	–	7.021E-02	–	–
1.800E+01	6.758E-02	–	–	6.679E-02	–	–	7.014E-02	–	–
2.000E+01	6.769E-02	–	–	6.689E-02	–	–	7.026E-02	–	–
2.200E+01	6.794E-02	–	–	6.713E-02	–	–	7.051E-02	–	–
2.400E+01	6.827E-02	–	–	6.746E-02	–	–	7.085E-02	–	–
2.600E+01	6.863E-02	–	–	6.781E-02	–	–	7.122E-02	–	–
2.800E+01	6.906E-02	–	–	6.823E-02	–	–	7.166E-02	–	–
3.000E+01	6.948E-02	–	–	6.865E-02	–	–	7.210E-02	–	–
4.000E+01	7.168E-02	–	–	7.081E-02	–	–	7.437E-02	–	–
5.000E+01	7.376E-02	–	–	7.287E-02	–	–	7.653E-02	–	–
6.000E+01	7.561E-02	–	–	7.469E-02	–	–	7.844E-02	–	–
8.000E+01	7.871E-02	–	–	7.774E-02	–	–	8.165E-02	–	–
1.000E+02	8.113E-02	–	–	8.013E-02	–	–	8.416E-02	–	–
1.500E+02	8.542E-02	–	–	8.437E-02	–	–	8.861E-02	–	–
2.000E+02	8.821E-02	–	–	8.712E-02	–	–	9.151E-02	–	–
3.000E+02	9.169E-02	–	–	9.055E-02	–	–	9.511E-02	–	–
4.000E+02	9.381E-02	–	–	9.265E-02	–	–	9.731E-02	–	–
5.000E+02	9.523E-02	–	–	9.405E-02	–	–	9.878E-02	–	–
6.000E+02	9.628E-02	–	–	9.509E-02	–	–	9.987E-02	–	–
8.000E+02	9.770E-02	–	–	9.649E-02	–	–	1.013E-01	–	–
1.000E+03	9.863E-02	–	–	9.741E-02	–	–	1.023E-01	–	–
1.500E+03	1.000E-01	–	–	9.879E-02	–	–	1.038E-01	–	–
2.000E+03	1.008E-01	–	–	9.954E-02	–	–	1.045E-01	–	–
3.000E+03	1.016E-01	–	–	1.004E-01	–	–	1.054E-01	–	–
4.000E+03	1.021E-01	–	–	1.009E-01	–	–	1.059E-01	–	–
5.000E+03	1.024E-01	–	–	1.011E-01	–	–	1.062E-01	–	–
6.000E+03	1.026E-01	–	–	1.013E-01	–	–	1.064E-01	–	–
8.000E+03	1.029E-01	–	–	1.016E-01	–	–	1.067E-01	–	–
1.000E+04	1.030E-01	–	–	1.018E-01	–	–	1.069E-01	–	–
1.500E+04	1.033E-01	–	–	1.020E-01	–	–	1.071E-01	–	–
2.000E+04	1.034E-01	–	–	1.021E-01	–	–	1.073E-01	–	–
3.000E+04	1.036E-01	–	–	1.023E-01	–	–	1.074E-01	–	–
4.000E+04	1.036E-01	–	–	1.023E-01	–	–	1.075E-01	–	–
5.000E+04	1.037E-01	–	–	1.024E-01	–	–	1.075E-01	–	–
6.000E+04	1.037E-01	–	–	1.024E-01	–	–	1.076E-01	–	–
8.000E+04	1.038E-01	–	–	1.025E-01	–	–	1.076E-01	–	–
1.000E+05	1.038E-01	–	–	1.025E-01	–	–	1.076E-01	–	–

**Table 4:** Single layers HVL/TVL for cementitious samples calculated using Py-AMA.Seidy and Py-MLBUF.

Photon Energy	HVL									HVL								
	B-C			Si-C			Al-C			B-C			Si-C			Al-C		
MeV	Py-AMA.seidy	Py-MLBUF	$\epsilon$	Py-AMA.seidy	Py-MLBUF	$\epsilon$	Py-AMA.seidy	Py-MLBUF	$\epsilon$	Py-AMA.seidy	Py-MLBUF	$\epsilon$	Py-AMA.seidy	Py-MLBUF	$\epsilon$	Py-AMA.seidy	Py-MLBUF	$\epsilon$
1.000E-03	5.532E-05	–	–	5.604E-05	–	–	5.338E-05	–	–	1.838E-04	–	–	1.862E-04	–	–	1.773E-04	–	–
1.500E-03	1.493E-04	–	–	1.513E-04	–	–	1.441E-04	–	–	4.961E-04	–	–	5.027E-04	–	–	4.788E-04	–	–
2.000E-03	2.432E-04	–	–	2.447E-04	–	–	2.331E-04	–	–	8.079E-04	–	–	8.128E-04	–	–	7.743E-04	–	–
3.000E-03	6.748E-04	–	–	6.794E-04	–	–	6.473E-04	–	–	2.242E-03	–	–	2.257E-03	–	–	2.150E-03	–	–
4.000E-03	1.178E-03	–	–	1.189E-03	–	–	1.132E-03	–	–	3.914E-03	–	–	3.949E-03	–	–	3.760E-03	–	–
5.000E-03	8.115E-04	–	–	8.234E-04	–	–	7.843E-04	–	–	2.696E-03	–	–	2.735E-03	–	–	2.605E-03	–	–
6.000E-03	1.322E-03	–	–	1.341E-03	–	–	1.278E-03	–	–	4.390E-03	–	–	4.455E-03	–	–	4.244E-03	–	–
8.000E-03	2.334E-03	–	–	2.371E-03	–	–	2.257E-03	–	–	7.754E-03	–	–	7.876E-03	–	–	7.498E-03	–	–
1.000E-02	4.307E-03	–	–	4.375E-03	–	–	4.165E-03	–	–	1.431E-02	–	–	1.454E-02	–	–	1.384E-02	–	–
1.500E-02	1.343E-02	1.340E-02	-2.245E-01	1.364E-02	1.360E-02	-3.253E-01	1.299E-02	1.300E-02	8.553E-02	4.461E-02	4.460E-02	-3.139E-02	4.533E-02	4.530E-02	-5.664E-02	4.315E-02	4.310E-02	-1.116E-01
2.000E-02	3.040E-02	3.040E-02	-1.373E-02	3.089E-02	3.090E-02	3.290E-02	2.941E-02	2.940E-02	-1.822E-02	1.010E-01	1.010E-01	-4.742E-04	1.026E-01	1.026E-01	-1.347E-02	9.768E-02	9.770E-02	1.793E-02
2.634E-02	6.626E-02	6.630E-02	5.751E-02	6.732E-02	6.740E-02	1.261E-01	6.408E-02	6.410E-02	3.183E-02	2.201E-01	2.203E-01	8.303E-02	2.236E-01	2.238E-01	8.226E-02	2.129E-01	2.131E-01	1.091E-01
3.000E-02	9.517E-02	9.520E-02	3.131E-02	9.667E-02	9.670E-02	2.647E-02	9.203E-02	9.200E-02	-3.009E-02	3.161E-01	3.162E-01	1.626E-02	3.211E-01	3.211E-01	-1.415E-02	3.057E-01	3.057E-01	-3.070E-03
4.000E-02	2.053E-01	2.053E-01	1.017E-02	2.084E-01	2.084E-01	-2.161E-02	1.984E-01	1.984E-01	-1.416E-02	6.819E-01	6.820E-01	1.137E-02	6.924E-01	6.924E-01	-5.699E-03	6.592E-01	6.591E-01	-9.688E-03
5.000E-02	3.537E-01	3.537E-01	7.150E-03	3.589E-01	3.589E-01	-1.194E-02	3.417E-01	3.417E-01	-5.600E-04	1.175E+00	1.175E+00	1.005E-02	1.192E+00	1.192E+00	-6.907E-03	1.135E+00	1.135E+00	-9.619E-03
5.954E-02	5.153E-01	5.149E-01	-8.722E-02	5.227E-01	5.222E-01	-1.032E-01	4.976E-01	4.971E-01	-1.095E-01	1.712E+00	1.710E+00	-9.077E-02	1.737E+00	1.735E+00	-1.038E-01	1.653E+00	1.651E+00	-1.053E-01
6.000E-02	5.234E-01	5.234E-01	6.394E-03	5.309E-01	5.308E-01	-1.088E-02	5.054E-01	5.053E-01	-1.465E-02	1.739E+00	1.739E+00	6.557E-03	1.763E+00	1.763E+00	-4.048E-03	1.679E+00	1.679E+00	-6.922E-03
8.000E-02	8.621E-01	8.622E-01	9.364E-03	8.735E-01	8.735E-01	-2.776E-03	8.316E-01	8.316E-01	-4.875E-03	2.864E+00	2.864E+00	7.045E-03	2.902E+00	2.902E+00	-2.921E-03	2.763E+00	2.763E+00	-5.433E-03
1.000E-01	1.144E+00	1.144E+00	8.259E-03	1.158E+00	1.158E+00	-3.152E-03	1.103E+00	1.103E+00	-3.769E-03	3.801E+00	3.801E+00	6.094E-03	3.848E+00	3.848E+00	-1.112E-03	3.664E+00	3.664E+00	-2.527E-03
1.500E-01	1.603E+00	1.603E+00	3.828E-03	1.622E+00	1.622E+00	-1.726E-04	1.544E+00	1.544E+00	-7.123E-04	5.326E+00	5.326E+00	6.695E-03	5.387E+00	5.387E+00	9.672E-04	5.129E+00	5.129E+00	1.265E-04
2.000E-01	1.883E+00	1.883E+00	8.698E-03	1.904E+00	1.904E+00	3.228E-03	1.813E+00	1.813E+00	1.142E-03	6.255E+00	6.255E+00	6.735E-03	6.324E+00	6.325E+00	1.348E-03	6.022E+00	6.022E+00	1.287E-03
3.000E-01	2.267E+00	2.267E+00	5.872E-03	2.292E+00	2.292E+00	4.055E-03	2.182E+00	2.182E+00	2.893E-03	7.531E+00	7.531E+00	5.298E-03	7.613E+00	7.613E+00	1.919E-03	7.249E+00	7.249E+00	1.799E-03
4.000E-01	2.567E+00	2.567E+00	4.828E-03	2.594E+00	2.595E+00	4.018E-03	2.470E+00	2.470E+00	1.839E-03	8.526E+00	8.527E+00	5.707E-03	8.618E+00	8.619E+00	2.365E-03	8.206E+00	8.207E+00	1.947E-03
5.000E-01	2.828E+00	2.828E+00	7.201E-03	2.858E+00	2.858E+00	1.784E-03	2.721E+00	2.722E+00	3.368E-03	9.393E+00	9.394E+00	5.622E-03	9.494E+00	9.495E+00	2.809E-03	9.040E+00	9.041E+00	1.960E-03
6.000E-01	3.067E+00	3.067E+00	7.496E-03	3.100E+00	3.100E+00	1.628E-03	2.951E+00	2.951E+00	1.238E-03	1.019E+01	1.019E+01	5.640E-03	1.030E+01	1.030E+01	3.101E-03	9.804E+00	9.804E+00	1.865E-03
6.620E-01	3.207E+00	3.206E+00	-3.123E-02	3.241E+00	3.240E+00	-3.292E-02	3.086E+00	3.085E+00	-3.617E-02	1.065E+01	1.065E+01	-3.097E-02	1.077E+01	1.076E+01	-3.429E-02	1.025E+01	1.025E+01	-3.469E-02
8.000E-01	3.502E+00	3.502E+00	7.049E-03	3.540E+00	3.540E+00	3.958E-03	3.370E+00	3.370E+00	1.691E-03	1.163E+01	1.163E+01	5.703E-03	1.176E+01	1.176E+01	2.862E-03	1.120E+01	1.120E+01	1.455E-03
1.000E+00	3.901E+00	3.901E+00	7.144E-03	3.942E+00	3.943E+00	3.040E-03	3.754E+00	3.754E+00	2.618E-03	1.296E+01	1.296E+01	5.529E-03	1.310E+01	1.310E+01	2.783E-03	1.247E+01	1.247E+01	2.215E-03
1.173E+00	4.225E+00	4.225E+00	4.809E-03	4.271E+00	4.271E+00	1.697E-03	4.066E+00	4.066E+00	7.785E-04	1.404E+01	1.404E+01	3.563E-03	1.419E+01	1.419E+01	1.513E-03	1.351E+01	1.351E+01	-6.163E-04
1.333E+00	4.509E+00	4.508E+00	-1.938E-02	4.558E+00	4.557E+00	-2.099E-02	4.340E+00	4.339E+00	-2.232E-02	1.498E+01	1.498E+01	-1.859E-02	1.514E+01	1.514E+01	-2.185E-02	1.442E+01	1.441E+01	-2.219E-02
1.500E+00	4.789E+00	4.789E+00	5.346E-03	4.840E+00	4.841E+00	3.146E-03	4.609E+00	4.609E+00	1.132E-03	1.591E+01	1.591E+01	6.112E-03	1.608E+01	1.608E+01	2.568E-03	1.531E+01	1.531E+01	2.003E-03
2.000E+00	5.535E+00	5.535E+00	5.301E-03	5.594E+00	5.595E+00	2.454E-03	5.327E+00	5.327E+00	1.661E-03	1.839E+01	1.839E+01	5.822E-03	1.858E+01	1.858E+01	2.849E-03	1.770E+01	1.770E+01	1.599E-03
2.506E+00	6.181E+00	6.182E+00	2.686E-03	6.248E+00	6.248E+00	-5.355E-04	5.949E+00	5.949E+00	-1.602E-03	2.053E+01	2.053E+01	2.206E-03	2.076E+01	2.076E+01	-7.231E-04	1.976E+01	1.976E+01	-2.345E-03
3.000E+00	6.721E+00	6.721E+00	5.377E-03	6.794E+00	6.794E+00	1.616E-03	6.469E+00	6.469E+00	2.115E-03	2.233E+01	2.233E+01	5.776E-03	2.257E+01	2.257E+01	2.436E-03	2.149E+01	2.149E+01	1.403E-03
4.000E+00	7.608E+00	7.608E+00	5.616E-03	7.691E+00	7.691E+00	1.869E-03	7.323E+00	7.323E+00	1.146E-03	2.527E+01	2.527E+01	5.501E-03	2.555E+01	2.555E+01	1.943E-03	2.433E+01	2.433E+01	8.336E-04
5.000E+00	8.280E+00	8.280E+00	5.194E-03	8.371E+00	8.372E+00	1.062E-03	7.971E+00	7.971E+00	2.062E-04	2.750E+01	2.751E+01	5.089E-03	2.781E+01	2.781E+01	1.346E-03	2.648E+01	2.648E+01	5.044E-04
6.000E+00	8.785E+00	8.785E+00	4.708E-03	8.883E+00	8.883E+00	8.047E-04	8.458E+00	8.458E+00	-5.027E-05	2.918E+01	2.918E+01	4.821E-03	2.951E+01	2.951E+01	9.686E-04	2.810E+01	2.810E+01	7.641E-05
8.000E+00	9.464E+00	9.465E+00	4.124E-03	9.572E+00	9.572E+00	4.552E-04	9.114E+00	9.114E+00	-1.737E-04	3.144E+01	3.144E+01	4.489E-03	3.180E+01	3.180E+01	-5.912E-05	3.028E+01	3.028E+01	-3.477E-04
1.000E+01	9.853E+00	9.853E+00	3.032E-03	9.966E+00	9.966E+00	-1.392E-03	9.489E+00	9.489E+00	-2.098E-03	3.273E+01	3.273E+01	3.162E-03	3.311E+01	3.311E+01	-1.402E-03	3.152E+01	3.152E+01	-1.806E-03
1.500E+01	1.023E+01	1.023E+01	3.614E-03	1.035E+01	1.035E+01	-1.619E-03	9.854E+00	9.854E+00	-1.694E-03	3.398E+01	3.398E+01	3.417E-03	3.438E+01	3.438E+01	-1.812E-03	3.273E+01	3.273E+01	-1.838E-03
1.600E+01	1.025E+01	–	–	1.037E+01	–	–	9.873E+00	–	–	3.404E+01	–	–	3.445E+01	–	–	3.280E+01	–	–
1.800E+01	1.026E+01	–	–	1.038E+01	–	–	9.882E+00	–	–	3.407E+01	–	–	3.448E+01	–	–	3.283E+01	–	–
2.000E+01	1.024E+01	–	–	1.036E+01	–	–	9.866E+00	–	–	3.402E+01	–	–	3.442E+01	–	–	3.277E+01	–	–
2.200E+01	1.020E+01	–	–	1.032E+01	–	–	9.831E+00	–	–	3.389E+01	–	–	3.430E+01	–	–	3.266E+01	–	–
2.400E+01	1.015E+01	–	–	1.028E+01	–	–	9.783E+00	–	–	3.373E+01	–	–	3.413E+01	–	–	3.250E+01	–	–
2.600E+01	1.010E+01	–	–	1.022E+01	–	–	9.733E+00	–	–	3.355E+01	–	–	3.396E+01	–	–	3.233E+01	–	–
2.800E+01	1.004E+01	–	–	1.016E+01	–	–	9.672E+00	–	–	3.334E+01	–	–	3.375E+01	–	–	3.213E+01	–	–
3.000E+01	9.976E+00	–	–	1.010E+01	–	–	9.614E+00	–	–	3.314E+01	–	–	3.354E+01	–	–	3.194E+01	–	–
4.000E+01	9.670E+00	–	–	9.789E+00	–	–	9.320E+00	–	–	3.212E+01	–	–	3.252E+01	–	–	3.096E+01	–	–
5.000E+01	9.397E+00	–	–	9.513E+00	–	–	9.057E+00	–	–	3.122E+01	–	–	3.160E+01	–	–	3.009E+01	–	–
6.000E+01	9.167E+00	–	–	9.281E+00	–	–	8.836E+00	–	–	3.045E+01	–	–	3.083E+01	–	–	2.935E+01	–	–
8.000E+01	8.807E+00	–	–	8.916E+00	–	–	8.489E+00	–	–	2.925E+01	–	–	2.962E+01	–	–	2.820E+01	–	–
1.000E+02	8.544E+00	–	–	8.650E+00	–	–	8.236E+00	–	–	2.838E+01	–	–	2.873E+01	–	–	2.736E+01	–	–
1.500E+02	8.114E+00	–	–	8.216E+00	–	–	7.822E+00	–	–	2.695E+01	–	–	2.729E+01	–	–	2.598E+01	–	–
2.000E+02	7.858E+00	–	–	7.956E+00	–	–	7.575E+00	–	–	2.610E+01	–	–	2.643E+01	–	–	2.516E+01	–	–
3.000E+02	7.560E+00	–	–	7.654E+00	–	–	7.288E+00	–	–	2.511E+01	–	–	2.543E+01	–	–	2.421E+01	–	–
4.000E+02	7.389E+00	–	–	7.482E+00	–	–	7.123E+00	–	–	2.455E+01	–	–	2.485E+01	–	–	2.366E+01	–	–
5.000E+02	7.278E+00	–	–	7.370E+00	–	–	7.017E+00	–	–	2.418E+01	–	–	2.448E+01	–	–	2.331E+01	–	–
6.000E+02	7.199E+00	–	–	7.289E+00	–	–	6.940E+00	–	–	2.391E+01	–	–	2.421E+01	–	–	2.306E+01	–	–
8.000E+02	7.095E+00	–	–	7.184E+00	–	–	6.840E+00	–	–	2.357E+01	–	–	2.386E+01	–	–	2.272E+01	–	–
1.000E+03	7.028E+00	–	–	7.116E+00	–	–	6.775E+00	–	–	2.335E+01	–	–	2.364E+01	–	–	2.251E+01	–	–
1.500E+03	6.930E+00	–	–	7.016E+00	–	–	6.680E+00	–	–	2.302E+01	–	–	2.331E+01	–	–	2.219E+01	–	–
2.000E+03	6.877E+00	–	–	6.963E+00	–	–	6.630E+00	–	–	2.285E+01	–	–	2.313E+01	–	–	2.202E+01	–	–
3.000E+03	6.819E+00	–	–	6.905E+00	–	–	6.574E+00	–	–	2.265E+01	–	–	2.294E+01	–	–	2.184E+01	–	–
4.000E+03	6.788E+00	–	–	6.873E+00	–	–	6.544E+00	–	–	2.255E+01	–	–	2.283E+01	–	–	2.174E+01	–	–
5.000E+03	6.769E+00	–	–	6.854E+00	–	–	6.526E+00	–	–	2.249E+01	–	–	2.277E+01	–	–	2.168E+01	–	–
6.000E+03	6.756E+00	–	–	6.841E+00	–	–	6.513E+00	–	–	2.244E+01	–	–	2.272E+01	–	–	2.164E+01	–	–
8.000E+03	6.738E+00	–	–	6.822E+00	–	–	6.496E+00	–	–	2.238E+01	–	–	2.266E+01	–	–	2.158E+01	–	–
1.000E+04	6.727E+00	–	–	6.811E+00	–	–	6.485E+00	–	–	2.235E+01	–	–	2.263E+01	–	–	2.154E+01	–	–
1.500E+04	6.711E+00	–	–	6.795E+00	–	–	6.469E+00	–	–	2.229E+01	–	–	2.257E+01	–	–	2.149E+01	–	–
2.000E+04	6.702E+00	–	–	6.786E+00	–	–	6.461E+00	–	–	2.226E+01	–	–	2.254E+01	–	–	2.146E+01	–	–
3.000E+04	6.693E+00	–	–	6.777E+00	–	–	6.453E+00	–	–	2.224E+01	–	–	2.251E+01	–	–	2.144E+01	–	–
4.000E+04	6.689E+00	–	–	6.773E+00	–	–	6.448E+00	–	–	2.222E+01	–	–	2.250E+01	–	–	2.142E+01	–	–
5.000E+04	6.686E+00	–	–	6.770E+00	–	–	6.446E+00	–	–	2.221E+01	–	–	2.249E+01	–	–	2.141E+01	–	–
6.000E+04	6.684E+00	–	–	6.768E+00	–	–	6.444E+00	–	–	2.220E+01	–	–	2.248E+01	–	–	2.141E+01	–	–
8.000E+04	6.680E+00	–	–	6.764E+00	–	–	6.440E+00	–	–	2.219E+01	–	–	2.247E+01	–	–	2.139E+01	–	–
1.000E+05	6.680E+00	–	–	6.763E+00	–	–	6.439E+00	–	–	2.219E+01	–	–	2.247E+01	–	–	2.139E+01	–	–

Table 5 compares the MAC and LAC of present versus published nano-modified cementitious shields. The B-C, Si-C, and Al-C samples exhibit markedly higher LAC values than those reported in the literature. Although the MAC values of the present samples (approximately 7.69-7.70 × 10$^{-2}$ cm$^{2}$/g) are slightly lower than those for cement with nano-PbO or nano-CdO (approximately 8.1-8.2 × 10$^{-2}$ cm$^{2}$/g), the LAC values of the present samples (2.14-2.25 × 10$^{-1}$ cm$^{-1}$) are substantially higher. This superior performance is primarily attributable to the higher physical density of the developed composites and the optimized nano-modifier content, which enhance photon interaction probability per unit thickness. Thus, despite comparable mass attenuation, the present materials offer superior linear attenuation, making them particularly effective for gamma-ray shielding applications where shielding thickness is constrained.

**Table 5:** Comparison of MAC and LAC of present versus published nano-modified cementitious shields.

Material	MAC (cm$^{2}$/g)	LAC (cm$^{-1}$)	Ref.
Cement + 2% Nano Fe$_{2}$O$_{3}$	$\sim$0.079 (at 0.662 MeV)	$\sim$0.183	^51^
Cement-Ball Clay + 16.7% Nano CdO	$\sim$0.082 (at 0.662 MeV)	$\sim$0.171	^52^
Mortar + 30% Nano Waste Glass	$\sim$0.077 (at 0.662 MeV)	$\sim$0.160	^53^
Cement + 0.5% Nano MnFe$_{2}$O$_{4}$	$\sim$0.078 (at 0.662 MeV)	$\sim$0.174	^54^
Concrete + 5% Nano PbO	$\sim$0.081 (at 0.662 MeV)	$\sim$0.198	^55^
Cement + 25% Nano Fe$_{2}$O$_{3}$	$\sim$0.080 (at 0.662 MeV)	$\sim$0.190	^56^
B-C	7.69E-02	2.16E-01	This study
Si-C	7.70E-02	2.14E-01	
Al-C	7.70E-02	2.25E-01	

### Microstructure characterization of aged cementitious materials

#### Morphological analysis-SEM

**Fig. 4:**
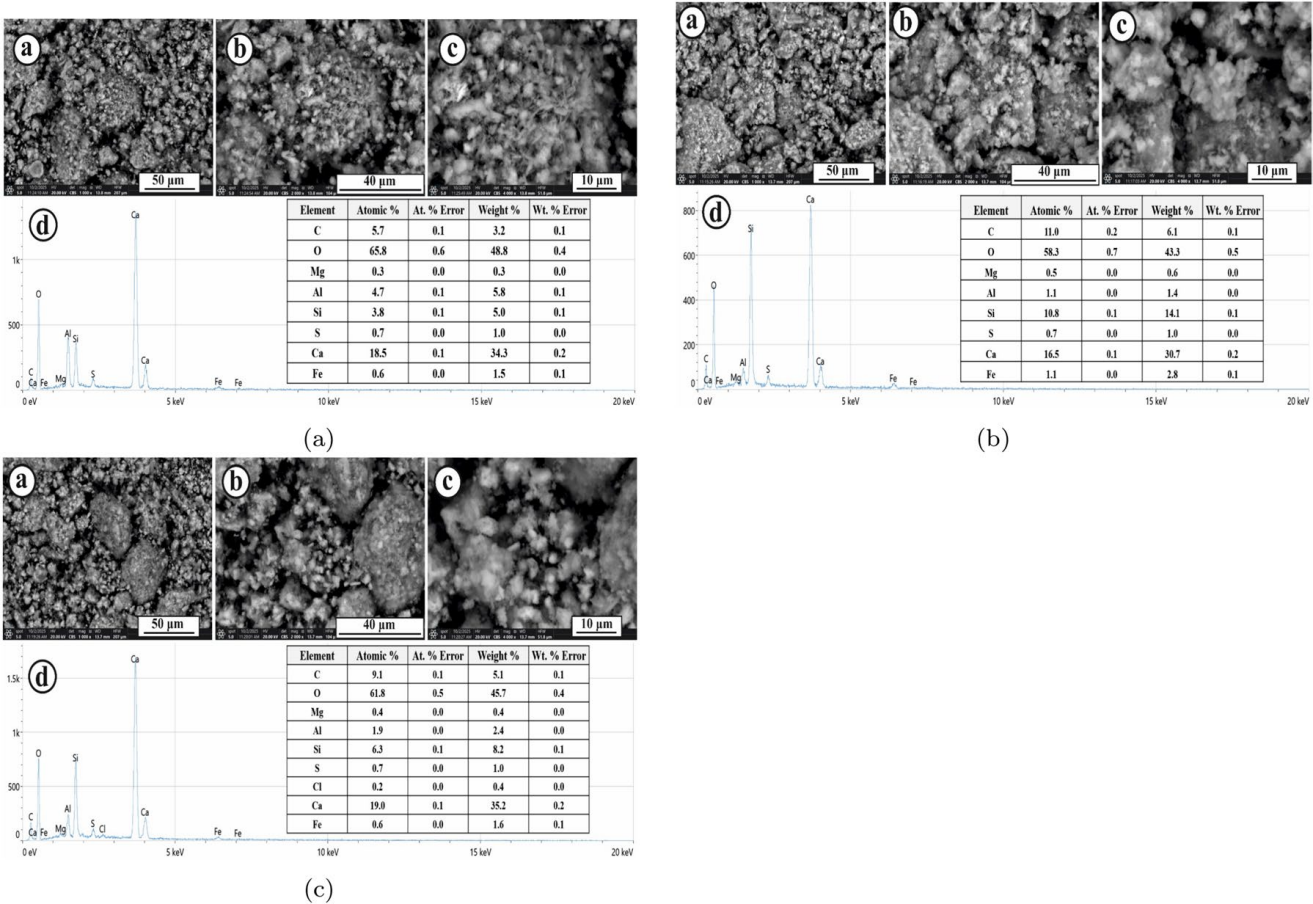
(**a**) SEM micrographs analysis for B-C (**a**–**c**) and EDX analysis (**d**), (**b**) SEM micrographs analysis for Si-C (**a**–**c**) and EDX analysis (**d**) and (**c**) SEM micrographs analysis for Al-C (**a**–**c**) and EDX analysis (**d**).

Figure 4 presents the SEM images (a-c) of the aged cementitious samples at different magnifications. These images reveal quasi-spherical particles with minor irregularities, uniformly distributed across all samples, with particle sizes ranging from approximately 0.5 to 50 $\mu$m. A detailed microstructural interpretation is outlined below.

The SEM micrographs of blank sample B-C (Fig. 4a(a–c)), representing aged hydrated ordinary Portland cement (OPC), exhibit a typical multiscale, heterogeneous microstructure of a partially hydrated cement paste. At low magnification ($\sim$1000×), the surface displays densely packed angular to sub-rounded grains interspersed with finer material. At medium magnification ($\sim$2000×), coarser particles appear increasingly coated and bridged by a finer continuous phase that fills voids and reduces visible porosity. At higher magnification ($\sim$4000×), the microstructure resolves into a network of amorphous/flocculent gel interspersed with acicular or needle-like crystals. The amorphous nodular phase is attributed to C-S-H gel, while the blocky or plate-like crystals that represent calcium hydroxide (CH) are invisible due to an amorphous substance that might be the Secondary C-S-H or due to the carbonation. The needle-like crystal represents the ettringite (AFt) formed from sulfate-aluminate reactions during hydration reaction.

For Si-C (5 wt% nano-silica), the SEM micrographs (Fig. 4b(a–c)) reveal significant microstructural changes compared to the blank sample. At low magnification, a heterogeneous mixture of angular and granular particles is noted. At medium magnification, the structure becomes denser compared to the blank sample (Fig. 4 a (b)), with intergranular voids filled. At the highest magnification, the matrix exhibits a compact, homogeneous, and globular morphology. The presented flocculent and nodular texture indicates secondary C-S-H gel development facilitated by nano-silica’s pozzolanic reactivity. The less amount of void refers to the potential role of nano-silica to act as nucleation sites for C-S-H formation and as a microfiller. Additionally, the presence of amorphous material can reflect the progress of the carbonation process.

Similarly, the Al-C sample containing 5 wt% nano-alumina (Figure 4c(a–c)) shows a markedly densified surface morphology than that of the blank sample. At low magnification, irregular agglomerates and hydrated grains are embedded in fine hydration products, indicating enhanced consolidation. Medium magnification reveals densely packed particles within a continuous C-S-H/C-A-S-H gel network, while high magnification displays a compact, homogeneous matrix with fewer pores. The cover of distinct CH plates with amorphous material suggests that nano-alumina consumes Ca(OH)₂ to form additional C-A-S-H and AFm phases or the progress of the carbonation process, resulting in a refined, low-porosity microstructure.

#### Elemental composition analysis-EDX

The EDX spectra (Fig. 4 and Table 6) confirm the presence of the major elements in all samples, including k$\alpha$ signals for Ca, Si, Al, Fe, C, and O, which were identified at energies of 3.692 keV, 1.740 keV, 1.487 keV, 6.398 keV, 0.277 keV, and 0.524 keV, respectively 53, 54. Additionally, S signals were detected at 2.308 keV and 2.816 keV for K$\alpha$ and K$\beta$, respectively 54-56. Furthermore, the presence of various element signals, including Na, Mg, and Cl, was observed as follows: in B-C, the Mg K$\alpha$ signal was detected at 1.254 keV; while in Si-C, the Na and Cl K$\alpha$ signals were detected at 1.041 keV and 2.622 keV, respectively 53,56. In contrast, these signals were absent in Al-C. The EDX technique was used for semi-quantitative surface analysis to compare and trace changes in the elemental composition of the raw sample (B-C, as a control) and after both modified samples 56. The EDX results reveal clear compositional differences among the three samples, reflecting their distinct hydration behaviors and microstructural evolution. For the blank sample B-C, oxygen (65.8 at%) and calcium (18.5 at%) dominate, with appreciable aluminum (4.7 at%) and relatively low silicon (3.8 at%), along with traces of sulfur, iron, and magnesium. The calculated atomic ratios, Ca/Si $\approx$ 4.87 and Ca/(Si+Al) $\approx$ 2.18, indicate a markedly calcium-rich composition, consistent with the presence of portlandite (Ca(OH)$_{2}$), carbonate phases (CaCO₃), and calcium-bearing crystalline hydrates coexisting with limited amounts of C-S-H and aluminous phases (AFt/AFm). In contrast, Si-C containing 5 wt% nano-silica, exhibits oxygen (58.3 at%), calcium (16.5 at%), and a significantly higher silicon content (10.8 at%), with minor aluminum, sulfur, magnesium, and iron. Its Ca/Si $\approx$ 1.53 and Ca/(Si+Al) $\approx$ 1.39 ratios fall within the range typical of C-S-H or C-A-S-H gels, indicating extensive pozzolanic consumption of Ca(OH)$_{2}$ and the formation of secondary silicate hydrates. This composition reflects the strong filler-pozzolanic dual role of nano-silica, which enhances hydration kinetics, promotes homogeneous C-S-H formation, and leads to a denser, low-porosity microstructure. For Al-C, incorporating 5 wt% nano-alumina, oxygen (61.8 at%) and calcium (19.0 at%) remain dominant, accompanied by moderate carbon (9.1 at%) and silicon (6.3 at%), smaller amounts of aluminum (1.9 at%), sulfur (0.7 at%), magnesium (0.4 at%), and traces of chlorine and iron. The computed Ca/Si $\approx$ 3.02 and Ca/(Si+Al) $\approx$ 2.32 suggest a Ca-rich environment where some portlandite persists due to the lower pozzolanic reactivity of nano-alumina compared to nano-silica. Nonetheless, the measurable aluminum and sulfur contents support the formation of secondary C-A-S-H and AFm/AFt phases, which aid in matrix densification and structural refinement. Overall, the quantitative comparison confirms that while B-C remains dominated by Ca-rich crystalline phases, Si-C shows significant silica enrichment and enhanced C-S-H formation, and Al-C demonstrates an intermediate state characterized by partial CH consumption and the emergence of Al-bearing hydrates. These trends collectively emphasize that nano-silica produces the most chemically balanced and microstructurally refined cement matrix among the studied samples.

Generally, the microstructural and compositional analyses of B-C (OPC), Si-C (nano-SiO₂), and Al-C (nano-Al₂O₃) reveal clear differences in hydration and densification. B-C exhibits a coarse, porous texture with loosely bound C-S-H gel, large Ca(OH)₂ crystals, and minor ettringite, indicating incomplete hydration. Si-C shows a dense, homogeneous morphology with fine, well-distributed C-S-H gel and minimal CH, confirming nano-silica’s strong pozzolanic and filler effects that enhance hydration, silica polymerization, and microstructural compactness. Al-C displays a moderately refined structure with needle- and plate-like crystals embedded in a cohesive matrix, suggesting the formation of C-A-S-H and AFt/AFm phases driven by nano-alumina’s nucleation activity. EDX data support these observations: B-C is Ca-rich (Ca/Si $\approx$ 4.9), Si-C has a balanced composition (Ca/Si $\approx$ 1.5-1.8) indicative of extensive silicate gel formation, and Al-C shows intermediate Ca/(Si+Al) $\approx$ 1.9-2.0, reflecting coexisting C-S-H and C-A-S-H phases. Overall, matrix refinement follows the order Si-C > Al-C > B-C, confirming that nano-silica achieves the greatest densification and gel evolution, while nano-alumina promotes aluminate hydrate formation and structural consolidation.

**Table 6:** Density and elemental composition of the investigated aged cementitious samples.

Element	B-C	Si-C	Al-C			
	Atomic %	Weight %	Atomic %	Weight %	Atomic %	Weight %
C	5.7 ±0.1	3.2 ±0.1	11.0 ±0.2	6.1 ±0.1	9.1 ±0.1	5.1 ±0.1
O	65.8 ±0.6	48.8 ±0.4	58.3 ±0.7	43.3 ±0.5	61.8 ±0.5	45.7 ±0.4
Mg	0.3 ±0.0	0.3 ±0.0	0.4 ±0.0	0.5 ±0.0	0.4 ±0.0	0.4 ±0.0
Al	4.7 ±0.1	5.8 ±0.1	1.1 ±0.0	1.4 ±0.0	1.9 ±0.0	2.4 ±0.0
Si	3.8 ±0.1	5.0 ±0.1	10.8 ±0.1	14.1 ±0.1	6.3 ±0.1	8.2 ±0.1
S	0.7 ±0.0	1.0 ±0.1	0.7 ±0.0	1.0 ±0.0	0.7 ±0.0	1.0 ±0.0
Cl	0.0 ±0.0	0.0 ±0.0	0.0 ±0.0	0.0 ±0.0	0.2 ±0.0	0.4 ±0.0
Ca	18.5 ±0.1	34.3 ±0.2	16.5 ±0.1	30.7 ±0.2	19.0 ±0.1	35.2 ±0.2
Fe	0.6 ±0.0	1.5 ±0.1	1.1 ±0.1	2.8 ±0.1	0.6 ±0.0	1.6 ±0.1

#### XRD

**Fig. 5:**
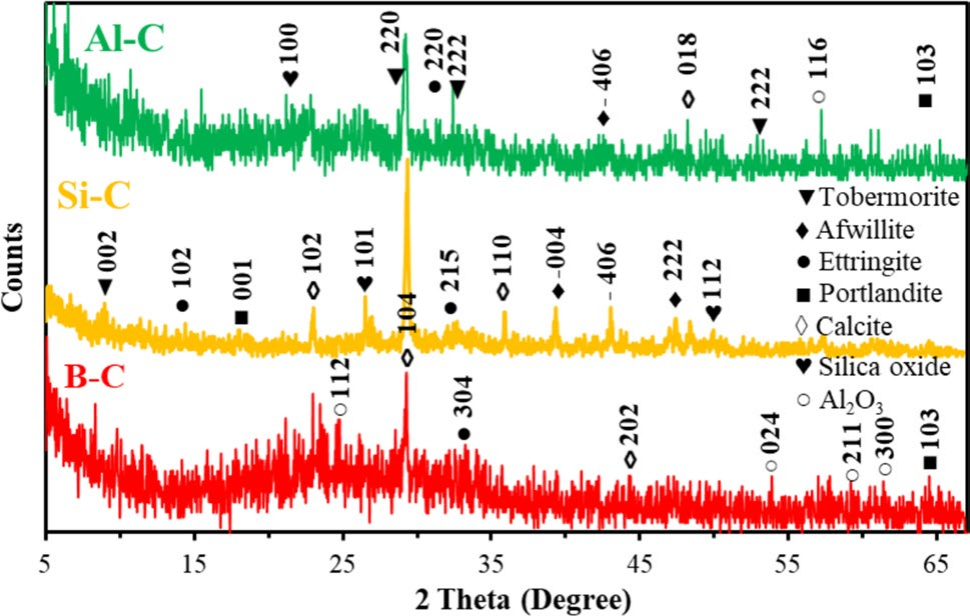
XRD patterns of B-C, Si-C, and Al-C samples.0.

The XRD phase composition provides clear evidence of the effect of nano-additive type on hydration reactions and phase evolution in B-C, Si-C, and Al-C matrices. Figure 5 displays the raw XRD pattern for the investigated aged cementitious samples prior to processing. Table 7 shows the XRD-identified phases of B-C, Si-C, and Al-C using Match software version 4.2. Si-C, containing nano-SiO₂, shows the highest tobermorite content (53.6%), attributed to the strong pozzolanic activity of silica, which consumes portlandite (Ca(OH)$_{2}$) and promotes the formation of additional calcium silicate hydrate (C-S-H) phases through secondary hydration reactions. This process enhances tobermorite crystallization and matrix densification ((PDF #97-008-7690) 57-59. The low portlandite content (3.1%) in Si-C, compared to B-C (10.3%) and Al-C (10.8%), confirms the effective transformation of Ca(OH)₂ into C-S-H. Such behavior aligns with the known mechanism where portlandite is consumed in pozzolanic reactions, generating secondary C-S-H with improved structural compactness 59,60. Afwillite and jennite, calcium silicate hydrate phases, decrease significantly in Si-C (8.8% and 1.7%) relative to B-C (13.9% and 4.4%), supporting a shift toward more stable tobermorite phases. Ettringite content slightly declines in Si-C (11.7%) and more notably in Al-C (7.8%), implying that the alumina additive promotes conversion to AFm or C-A-S-H phases. Notably, calcite content increases substantially in Al-C (14.9%), compared with B-C (7.5%) and Si-C (9.3%), which may result from early carbonation of this sample that allows the amorphous calcium carbonate to transform into the more stable calcite 12. The higher residual silica oxide in Al-C (12.4%) and lower in Si-C (6.4%) indicates that nano-silica is more reactive, while part of the added alumina may be incorporated into poorly crystalline C-A-S-H phases, leading to a lower detectable alumina oxide fraction (2.3%). Overall, Si-C exhibits the most refined and densified microstructure with the most efficient formation of C-S-H and tobermorite, followed by Al-C and B-C. These results confirm that nano-silica enhances hydration kinetics and matrix densification, whereas nano-alumina promotes aluminate and carbonate phase development, balancing strength and phase stability.

**Table 7:** XRD-identified phases of B-C, Si-C, and Al-C using Match software.

Phase	B-C	Si-C	Al-C
Tobermorite	37.70%	53.60%	35.50%
Afwillite	13.90%	8.80%	12.70%
Jennite	4.40%	1.70%	3.60%
Ettringite	12.80%	11.70%	7.80%
Portlandite	10.30%	3.10%	10.80%
Calcite	7.50%	9.30%	14.90%
Silica oxide	8.80%	6.40%	12.40%
Alumina oxide	4.70%	5.20%	2.30%

#### Dynamic light scattering (DLS) and zeta-potential analysis

**Fig. 6:**
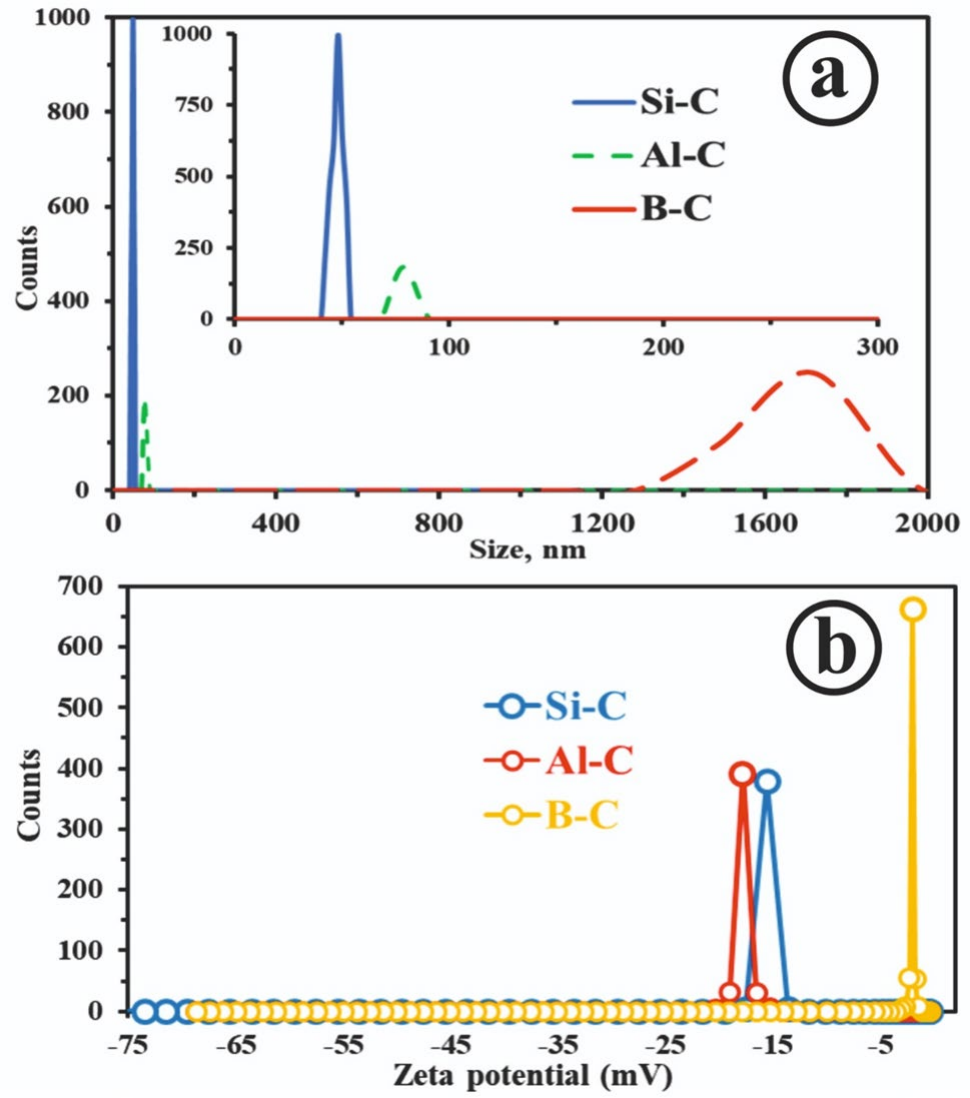
DLS and zeta-potential analysis for the B-C, Si-C, and Al-C samples.

The DLS analysis revealed z-average particle sizes of 1718.5 ± 35.2 nm for B-C, 48.4 ± 2.1 nm for Si-C, and 78.8 ± 3.5 nm for Al-C, with polydispersity indices of 0.42, 0.18, and 0.22, respectively (Fig. 6). Zeta-potential measurements showed values of -2.01 ± 0.3 mV (B-C), -15.56 ± 0.5 mV (Si-C), and -17.78 ± 0.5 mV (Al-C). The dramatic size reduction from $\sim$1.7 $\mu$m (B-C) to $\sim$48-79 nm (Si-C, Al-C) confirms the effective dispersion of the nano-modifier. The more negative zeta-potential values of Si-C (-15.56 mV) and Al-C (-17.78 mV) compared to B-C (-2.01 mV) indicate enhanced electrostatic repulsion and colloidal stability, which prevents nanoparticle agglomeration and promotes uniform distribution within the cement matrix. The smallest particle size of Si-C (48.4 nm) combined with its moderately negative zeta-potential (-15.56 mV) correlates with its highest tobermorite content (53.6%) and densest C-S-H matrix, while Al-C exhibits the most negative zeta-potential (-17.78 mV) and intermediate particle size (78.8 nm), which, together with its highest density (2.92 g·cm$^{-3}$) explains its superior gamma-ray attenuation. Finally, the coarse particle size of B-C (1718.5 nm) explains its higher porosity and inferior shielding performance.

#### Textural properties analysis

The BET surface area analysis revealed values of 94.64 m$^{2}$/g for B-C, 290.11 m$^{2}$/g for Si-C, and 416.07 m$^{2}$/g for Al-C, with corresponding average pore diameters of 28.82 nm, 9.40 nm, and 6.56 nm, and total pore volumes of 0.40, 0.56, and 1.45 cm$^{3}$/g, respectively (Table8).This 4.4-fold increase in surface area for Al-C and 3.1-fold increase for Si-C compared to B-C confirms that the nano-modifiers create a highly developed internal surface network. Integrating these results with DLS and zeta-potential measurements shows that Si-C and Al-C exhibit dramatically smaller particle sizes (48.4 nm and 78.8 nm, respectively) compared to B-C (1718.5 nm), with corresponding zeta-potentials of -15.56 mV (Si-C), -17.78 mV (Al-C), and -2.01 mV (B-C). The highly negative zeta-potentials of Si-C and Al-C provide strong electrostatic repulsion, preventing nanoparticle agglomeration and promoting uniform dispersion within the cement matrix, as confirmed by their low polydispersity indices (0.18 and 0.22, respectively) compared to B-C (0.42). The dramatic reduction in average pore diameter from B-C (28.82 nm) to Si-C (9.40 nm) and Al-C (6.56 nm) confirms that nano-modifiers effectively refine the pore structure, while the increased pore volume of Al-C (1.45 cm$^{3}$/g) indicates a highly developed mesoporous network. These structural features directly translate to macroscopic properties: the refined pore structure and high surface area of Si-C promote extensive pozzolanic reactions, evidenced by its highest tobermorite content (53.6%) and densest C-S-H matrix, while the combination of excellent dispersion, most negative zeta-potential, highest surface area, and smallest pore diameter of Al-C yields the highest bulk density (2.92 g·cm$^{-3}$) and, consequently, the superior gamma-ray attenuation (LAC = 53.37 cm$^{-1}$ at 0.015 MeV). Thus, the integrated BET-DLS-zeta-potential analysis quantitatively establishes the structure-property relationships that underpin the observed microstructural refinement and radiation shielding performance of the nano-modified cement composites.

**Table 8:** BET surface area, porosity, DLS particle size, and zeta-potential of B-C, Si-C, and Al-C.

Parameter	B-C	Si-C	Al-C
BET surface area (m$^{2}$/g)	94.64	290.11	416.07
Average pore diameter (nm)	28.82	9.4	6.56
Total pore volume (cm$^{3}$/g)	0.4	0.56	1.45
DLS particle size (nm)	1718.5	48.4	78.8
Zeta-potential (mV)	-2.01	-15.56	-17.78
Polydispersity index (PDI)	0.42	0.18	0.22

#### FTIR

**Fig. 7:**
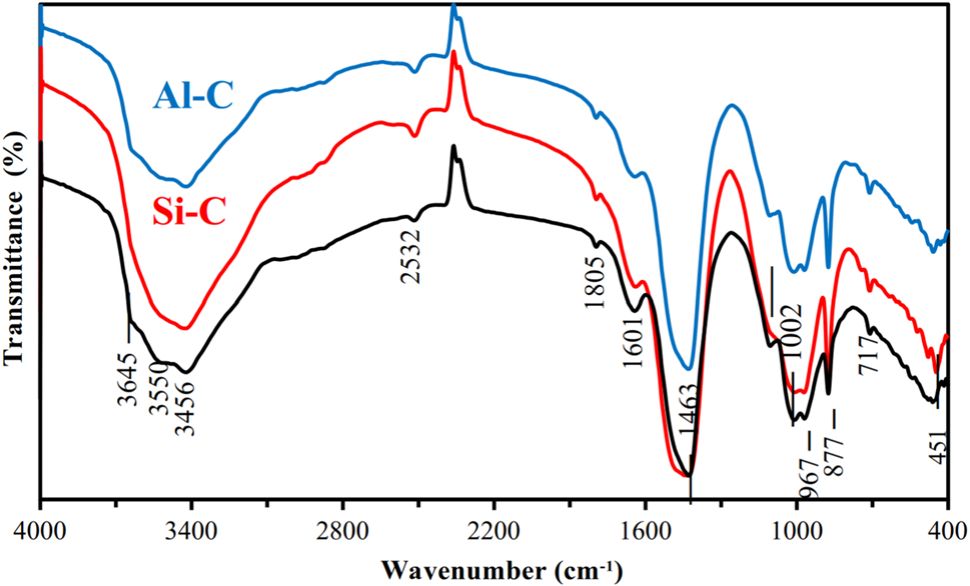
FTIR spectra of B-C, Si-C, and Al-C samples.

The FTIR spectra (Fig. 7) of the three cementitious samples (B-C, Si-C, and Al-C) exhibit characteristic vibrational bands corresponding to their hydration products and constituent phases. For the B-C spectrum, a medium-intensity H-O-H stretching vibration is centered at 3421 cm$^{-1}$, accompanied by a weak bending vibration at 1642 cm$^{-1}$, both attributed to the absorbed and bound water molecules in the hydrated phases^5,35^.

The stretching and bending vibrations of the carbonate group (CO$_{3}^{2-}$) from CaCO$_{3}$ appear at approximately 1427 cm$^{-1}$ and 875 cm$^{-1}$, respectively, indicating partial carbonation of hydration products^9,57^. The strong band at 1000 cm$^{-1}$ corresponds to the asymmetric stretching of Si-O bonds in silicate units, confirming the presence of calcium silicate hydrate (C-S-H) gel as the primary hydration product. Additionally, a weak band near 461 cm$^{-1}$ is associated with in-plane Si-O bending vibrations ($\nu _{4}$, $\nu _{2}$)^9,35^. For Si-C (cement with 5 wt% nano-silica), the spectrum shows a medium H-O-H stretching band at 3423 cm$^{-1}$^58,59^. The carbonate-related bands of CaCO$_{3}$ are observed at 1434 cm$^{-1}$ (stretching) and 875 cm$^{-1}$ (bending)^9,16,35^, indicating similar but slightly shifted features due to nano-silica incorporation. The distinct band at 971 cm$^{-1}$ corresponds to the stretching vibration of Si-O-Si and Si-O-Al ($\nu _{3}$) groups^9,16^, reflecting the formation of additional silicate and aluminosilicate networks. Bands in the region 416-796 cm$^{-1}$ arise from Si-O-Al, Si-O-Mg, and Si-O bending modes, confirming structural modification and densification of the silicate network due to the pozzolanic activity of nano-silica^58,59^.

For Al-C (cement with 5 wt% nano-alumina), a medium H-O-H stretching vibration appears at 3420 cm$^{-1}$, and the carbonate bands of CaCO$_{3}$ are detected at 1429 cm$^{-1}$ and 875 cm$^{-1}$^58,59^. The region between 416 and 796 cm$^{-1}$ exhibits bands assigned to Si-O-Al, Si-O-Mg, and Si-O bending and stretching modes^9,16^, confirming enhanced aluminosilicate network formation. A broad asymmetric Si-O-Si stretching band extends from 950 to 1250 cm$^{-1}$, with a sharp peak at 1012-1020 cm$^{-1}$, attributed to Q$^{2}$ silicate tetrahedral units characteristic of polymerized C-(A)-S-H gel structures^58–60^. These spectral features collectively confirm that nano-silica and nano-alumina incorporation promote additional C-S-H/C-A-S-H gel formation, matrix densification, and reduced portlandite content through secondary pozzolanic reactions.

## Conclusion

This study systematically evaluated the microstructure and gamma-ray shielding performance of two-year-aged cementitious composites modified with 5 wt % nano-silica (Si-C) and 5 wt% nano-alumina (Al-C), compared to unmodified ordinary Portland cement (B-C). Comprehensive characterization confirmed nano-modifier enhancement of cementitious composites. Density values were 2.78 (Si-C), 2.81 (B-C), and 2.92 g·cm$^{-3}$ (Al-C). BET analysis showed Si-C and Al-C have 3-4-fold higher surface areas (290-416 m$^{2}$/g) and smaller pores (6-9 nm) than B-C (95 m²/g, 29 nm). DLS/zeta-potential confirmed excellent dispersion (48-79 nm particles, -15.6 to -17.8 mV) versus B-C (1718 nm, -2.0 mV). These structure-property correlations underpin the shielding performance. Microstructural analysis (XRD, SEM-EDS, FTIR) further revealed that Si-C exhibited the highest tobermorite content (53.6%) and a dense C-S-H-rich matrix, while Al-C promoted C-A-S-H formation. Gamma-ray shielding parameters evaluated over 0.015-15 MeV using Py-MLBUF and Py-AMA.Seidy models, validated against NIST XCOM $(differences <0.4 \%)$ showed that Al-C exhibited the highest linear attenuation coefficient (53.37 cm⁻¹ at 0.015 MeV) and the lowest HVL/TVL values. Effective atomic numbers ranged from $\sim$11.8 to $\sim$17.3, with Al-C highest. Buildup factors at 1 mfp were lowest for Al-C, indicating reduced secondary radiation. For double-layer configurations, placing B-C as the first layer followed by nano-modified cement reduced buildup factors by 15-25% compared to the reverse order. Regarding applications, Al-C is recommended for medical radiotherapy and industrial radiography where space is limited, while Si-C is recommended for long-term engineered barriers in nuclear waste storage. Limitations include SEM-EDS surface sensitivity; future studies should employ XRF or ICP-OES. This work establishes quantitative structure-density-shielding relationships, offering design flexibility for radiation-shielding applications from medical facilities to nuclear waste repositories.

## Supplementary Information

Supplementary Information.

## Data Availability

All data generated or analysed during this study are included in this published article.
