# Supplementary material for: Radiation shielding and microstructural characteristics of nano-silica and nano-alumina modified cement composites
Source: Sci Rep. 2026 Jun 22;16:19364. doi: 10.1038/s41598-026-56740-x (PMC13287789; doi:10.1038/s41598-026-56740-x)
Supplement: Supplementary file 1 — Supplementary Information. [file 41598_2026_56740_MOESM1_ESM.pdf]

Appendix A. Supplementary Information

Table S1: Photoelectric Absorption of cementitious samples calculated using XCOM, Py-AMA.Seidy and Py-MLBUF.

Photon Energy	B-C					Si-C					Al-C				
MeV	XCOM	Py-AMA.seidy	Py-MLBUF	ϵ (a, b)	ϵ (a, c)	XCOM	Py-AMA.seidy	Py-MLBUF	ϵ (a, b)	ϵ (a, c)	XCOM	Py-AMA.seidy	Py-MLBUF	ϵ (a, b)	ϵ (a, c)
1.000E-03	4.456E+03	4.456E+03	-	-3.022E-03	-	4.446E+03	4.446E+03	-	4.946E-03	-	4.444E+03	4.444E+03	-	-6.682E-03	-
1.500E-03	1.649E+03	1.649E+03	-	9.249E-03	-	1.645E+03	1.645E+03	-	-6.244E-03	-	1.644E+03	1.644E+03	-	2.302E-02	-
2.000E-03	1.012E+03	1.012E+03	-	3.557E-04	-	1.017E+03	1.017E+03	-	-2.945E-02	-	1.016E+03	1.016E+03	-	5.000E-03	-
3.000E-03	3.636E+02	3.636E+02	-	1.076E-02	-	3.651E+02	3.651E+02	-	-7.013E-03	-	3.648E+02	3.648E+02	-	6.344E-03	-
4.000E-03	2.078E+02	2.077E+02	-	-2.543E-02	-	2.082E+02	2.082E+02	-	-2.152E-02	-	2.081E+02	2.081E+02	-	2.077E-03	-
5.000E-03	3.026E+02	3.026E+02	-	3.547E-03	-	3.015E+02	3.014E+02	-	-1.869E-02	-	3.013E+02	3.013E+02	-	-4.346E-03	-
6.000E-03	1.855E+02	1.855E+02	-	-3.156E-03	-	1.848E+02	1.848E+02	-	-2.184E-02	-	1.847E+02	1.847E+02	-	-2.045E-02	-
8.000E-03	1.047E+02	1.047E+02	-	3.364E-02	-	1.042E+02	1.042E+02	-	2.251E-02	-	1.042E+02	1.042E+02	-	2.832E-02	-
1.000E-02	5.652E+01	5.652E+01	-	-2.881E-03	-	5.624E+01	5.624E+01	-	-7.521E-03	-	5.624E+01	5.624E+01	-	1.268E-04	-
1.500E-02	1.785E+01	1.785E+01	1.785E+01	-1.242E-02	-2.241E-02	1.775E+01	1.776E+01	1.776E+01	3.058E-02	4.507E-02	1.776E+01	1.776E+01	1.776E+01	-1.502E-02	-5.631E-03
2.000E-02	7.710E+00	7.712E+00	7.711E+00	2.060E-02	1.297E-02	7.669E+00	7.671E+00	7.672E+00	2.430E-02	3.651E-02	7.670E+00	7.672E+00	7.673E+00	2.405E-02	3.520E-02
2.634E-02	3.402E+00	3.402E+00	3.405E+00	-9.241E-03	8.230E-02	3.383E+00	3.383E+00	3.387E+00	1.246E-02	1.242E-01	3.384E+00	3.384E+00	3.388E+00	-2.533E-03	1.064E-01
3.000E-02	2.300E+00	2.300E+00	2.300E+00	1.487E-02	4.348E-03	2.288E+00	2.288E+00	2.288E+00	-4.192E-03	8.741E-03	2.288E+00	2.288E+00	2.289E+00	1.117E-02	2.185E-02
4.000E-02	9.600E-01	9.599E-01	9.598E-01	-9.557E-03	-1.875E-02	9.547E-01	9.546E-01	9.548E-01	-6.072E-03	6.285E-03	9.549E-01	9.548E-01	9.549E-01	-9.854E-03	1.047E-03
5.000E-02	4.834E-01	4.834E-01	4.834E-01	3.888E-03	-6.206E-03	4.808E-01	4.807E-01	4.808E-01	-1.229E-02	0.000E+00	4.809E-01	4.808E-01	4.809E-01	-1.471E-02	-4.159E-03
5.954E-02	2.816E-01	2.816E-01	2.816E-01	1.147E-02	1.420E-02	2.800E-01	2.801E-01	2.801E-01	2.183E-02	4.643E-02	2.801E-01	2.801E-01	2.802E-01	5.391E-03	2.856E-02
6.000E-02	2.749E-01	2.749E-01	2.749E-01	1.495E-02	3.638E-03	2.734E-01	2.734E-01	2.734E-01	2.414E-03	1.463E-02	2.735E-01	2.735E-01	2.735E-01	-1.475E-02	-3.656E-03
8.000E-02	1.121E-01	1.121E-01	1.121E-01	4.825E-03	-8.921E-03	1.115E-01	1.115E-01	1.115E-01	-2.386E-02	-8.969E-03	1.115E-01	1.115E-01	1.115E-01	-3.091E-03	8.969E-03
1.000E-01	5.574E-02	5.574E-02	5.574E-02	7.915E-03	-3.588E-03	5.543E-02	5.543E-02	5.544E-02	-3.825E-03	9.020E-03	5.544E-02	5.544E-02	5.545E-02	-3.231E-05	1.082E-02
1.500E-01	1.566E-02	1.566E-02	1.566E-02	1.783E-03	-6.386E-03	1.557E-02	1.557E-02	1.557E-02	2.335E-03	1.285E-02	1.558E-02	1.557E-02	1.558E-02	-3.824E-02	-2.567E-02
2.000E-01	6.419E-03	6.419E-03	6.418E-03	-7.584E-03	-1.869E-02	6.382E-03	6.381E-03	6.382E-03	-9.225E-03	3.134E-03	6.384E-03	6.383E-03	6.384E-03	-1.577E-02	-4.699E-03
3.000E-01	1.889E-03	1.889E-03	1.889E-03	1.533E-02	5.294E-03	1.878E-03	1.878E-03	1.879E-03	1.537E-02	2.662E-02	1.879E-03	1.879E-03	1.879E-03	-1.190E-02	0.000E+00
4.000E-01	8.277E-04	8.279E-04	8.278E-04	1.828E-02	6.041E-03	8.229E-04	8.230E-04	8.231E-04	1.342E-02	2.673E-02	8.231E-04	8.232E-04	8.233E-04	1.583E-02	2.673E-02
5.000E-01	4.522E-04	4.522E-04	4.522E-04	4.272E-03	-8.846E-03	4.496E-04	4.496E-04	4.496E-04	-6.934E-03	6.673E-03	4.497E-04	4.497E-04	4.497E-04	-1.956E-03	8.895E-03
6.000E-01	2.838E-04	2.839E-04	2.838E-04	2.437E-02	1.057E-02	2.822E-04	2.822E-04	2.822E-04	1.109E-03	1.417E-02	2.823E-04	2.823E-04	2.823E-04	-6.739E-03	3.542E-03
6.620E-01	2.225E-04	2.224E-04	2.226E-04	-2.697E-02	2.247E-02	2.212E-04	2.211E-04	2.213E-04	-3.022E-02	4.521E-02	2.212E-04	2.212E-04	2.214E-04	-2.554E-03	7.233E-02
8.000E-01	1.442E-04	1.442E-04	1.442E-04	6.272E-03	-6.935E-03	1.434E-04	1.434E-04	1.434E-04	-2.664E-02	-1.395E-02	1.434E-04	1.434E-04	1.434E-04	1.113E-03	1.395E-02
1.000E+00	8.968E-05	8.967E-05	8.966E-05	-8.583E-03	-2.119E-02	8.915E-05	8.915E-05	8.916E-05	-5.137E-03	7.852E-03	8.918E-05	8.917E-05	8.918E-05	-1.100E-02	0.000E+00
1.173E+00	6.419E-05	6.418E-05	6.400E-05	-1.646E-02	-2.991E-01	6.381E-05	6.380E-05	6.364E-05	-1.355E-02	-2.696E-01	6.383E-05	6.382E-05	6.365E-05	-1.697E-02	-2.757E-01
1.333E+00	5.132E-05	5.132E-05	5.134E-05	-8.664E-03	3.313E-02	5.102E-05	5.101E-05	5.105E-05	-1.196E-02	5.684E-02	5.103E-05	5.103E-05	5.106E-05	-3.719E-03	6.271E-02
1.500E+00	4.153E-05	4.153E-05	4.152E-05	-6.871E-03	-1.926E-02	4.128E-05	4.128E-05	4.129E-05	7.371E-03	2.180E-02	4.130E-05	4.129E-05	4.130E-05	-1.337E-02	-2.421E-03
2.000E+00	2.572E-05	2.571E-05	2.571E-05	-2.049E-02	-3.110E-02	2.557E-05	2.556E-05	2.557E-05	-2.376E-02	-1.173E-02	2.557E-05	2.557E-05	2.557E-05	3.595E-03	1.564E-02
2.506E+00	1.822E-05	1.822E-05	1.824E-05	-1.199E-02	8.782E-02	1.811E-05	1.811E-05	1.813E-05	6.494E-03	1.270E-01	1.812E-05	1.812E-05	1.814E-05	-2.166E-02	9.934E-02
3.000E+00	1.405E-05	1.406E-05	1.405E-05	3.983E-02	2.847E-02	1.397E-05	1.397E-05	1.398E-05	2.470E-02	3.579E-02	1.398E-05	1.398E-05	1.398E-05	-2.009E-02	-7.153E-03
4.000E+00	9.506E-06	9.506E-06	9.505E-06	-2.592E-03	-1.368E-02	9.451E-06	9.450E-06	9.452E-06	-7.049E-03	6.349E-03	9.453E-06	9.453E-06	9.454E-06	-1.782E-03	1.058E-02
5.000E+00	7.139E-06	7.140E-06	7.139E-06	1.658E-02	5.603E-03	7.098E-06	7.099E-06	7.100E-06	8.789E-03	2.254E-02	7.099E-06	7.100E-06	7.101E-06	2.075E-02	3.240E-02
6.000E+00	5.699E-06	5.699E-06	5.699E-06	3.633E-03	-7.019E-03	5.666E-06	5.666E-06	5.667E-06	1.279E-03	1.588E-02	5.668E-06	5.668E-06	5.668E-06	-8.083E-03	5.293E-03
8.000E+00	4.047E-06	4.046E-06	4.046E-06	-2.177E-02	-2.965E-02	4.023E-06	4.023E-06	4.023E-06	-9.031E-03	7.457E-03	4.024E-06	4.024E-06	4.024E-06	-8.328E-03	7.455E-03
1.000E+01	3.130E-06	3.130E-06	3.130E-06	1.564E-02	0.000E+00	3.112E-06	3.112E-06	3.113E-06	1.089E-02	2.249E-02	3.113E-06	3.113E-06	3.113E-06	4.187E-03	1.285E-02
1.500E+01	1.994E-06	1.994E-06	1.993E-06	-1.496E-02	-3.009E-02	1.982E-06	1.982E-06	1.982E-06	8.067E-03	1.514E-02	1.983E-06	1.983E-06	1.983E-06	-1.722E-02	-1.009E-02
1.600E+01	1.858E-06	1.859E-06	-	3.251E-02	-	1.848E-06	1.848E-06	-	-8.331E-03	-	1.848E-06	1.848E-06	-	1.679E-02	-
1.800E+01	1.636E-06	1.636E-06	-	-2.710E-02	-	1.626E-06	1.626E-06	-	5.745E-03	-	1.627E-06	1.627E-06	-	-3.062E-02	-
2.000E+01	1.461E-06	1.461E-06	-	-2.624E-02	-	1.452E-06	1.452E-06	-	1.159E-02	-	1.453E-06	1.453E-06	-	-3.223E-02	-
2.200E+01	1.320E-06	1.319E-06	-	-3.845E-02	-	1.312E-06	1.312E-06	-	-1.054E-02	-	1.312E-06	1.312E-06	-	1.443E-02	-
2.400E+01	1.203E-06	1.203E-06	-	1.044E-02	-	1.196E-06	1.196E-06	-	1.413E-02	-	1.197E-06	1.196E-06	-	-4.446E-02	-
2.600E+01	1.105E-06	1.105E-06	-	2.028E-02	-	1.099E-06	1.099E-06	-	-1.502E-02	-	1.099E-06	1.099E-06	-	9.915E-03	-
2.800E+01	1.023E-06	1.023E-06	-	-4.514E-02	-	1.017E-06	1.017E-06	-	-3.663E-02	-	1.017E-06	1.017E-06	-	-1.175E-02	-
3.000E+01	9.510E-07	9.510E-07	-	-4.865E-03	-	9.455E-07	9.455E-07	-	-4.443E-03	-	9.458E-07	9.457E-07	-	-1.130E-02	-
4.000E+01	7.048E-07	7.049E-07	-	1.804E-02	-	7.007E-07	7.009E-07	-	2.207E-02	-	7.009E-07	7.010E-07	-	1.832E-02	-
5.000E+01	5.598E-07	5.598E-07	-	-4.689E-03	-	5.566E-07	5.565E-07	-	-1.060E-02	-	5.567E-07	5.567E-07	-	-3.840E-03	-
6.000E+01	4.642E-07	4.642E-07	-	-4.467E-03	-	4.615E-07	4.615E-07	-	-2.261E-04	-	4.616E-07	4.616E-07	-	2.845E-03	-
8.000E+01	3.461E-07	3.461E-07	-	-3.805E-03	-	3.441E-07	3.441E-07	-	-3.238E-03	-	3.442E-07	3.442E-07	-	-7.614E-03	-
1.000E+02	2.758E-07	2.759E-07	-	2.351E-02	-	2.742E-07	2.743E-07	-	2.642E-02	-	2.743E-07	2.743E-07	-	1.462E-02	-
1.500E+02	1.830E-07	1.830E-07	-	8.076E-03	-	1.819E-07	1.820E-07	-	3.216E-02	-	1.820E-07	1.820E-07	-	1.827E-03	-
2.000E+02	1.369E-07	1.369E-07	-	-1.779E-02	-	1.361E-07	1.361E-07	-	-1.044E-02	-	1.361E-07	1.361E-07	-	1.421E-02	-
3.000E+02	9.104E-08	9.102E-08	-	-2.101E-02	-	9.051E-08	9.050E-08	-	-1.578E-02	-	9.054E-08	9.052E-08	-	-2.431E-02	-
4.000E+02	6.820E-08	6.820E-08	-	1.372E-03	-	6.780E-08	6.781E-08	-	1.096E-02	-	6.782E-08	6.782E-08	-	6.026E-03	-
5.000E+02	5.452E-08	5.452E-08	-	4.490E-03	-	5.421E-08	5.421E-08	-	-3.931E-03	-	5.422E-08	5.422E-08	-	2.202E-03	-
6.000E+02	4.540E-08	4.540E-08	-	1.024E-02	-	4.514E-08	4.514E-08	-	5.998E-03	-	4.515E-08	4.515E-08	-	8.382E-03	-
8.000E+02	3.403E-08	3.403E-08	-	1.009E-02	-	3.384E-08	3.384E-08	-	-8.638E-03	-	3.384E-08	3.385E-08	-	1.594E-02	-
1.000E+03	2.722E-08	2.722E-08	-	6.580E-03	-	2.706E-08	2.706E-08	-	1.751E-02	-	2.707E-08	2.707E-08	-	5.143E-03	-
1.500E+03	1.814E-08	1.814E-08	-	-1.641E-02	-	1.803E-08	1.803E-08	-	1.322E-02	-	1.804E-08	1.804E-08	-	-1.764E-02	-
2.000E+03	1.360E-08	1.360E-08	-	-1.101E-02	-	1.352E-08	1.352E-08	-	3.670E-04	-	1.352E-08	1.352E-08	-	2.499E-02	-
3.000E+03	9.064E-09	9.063E-09	-	-9.115E-03	-	9.011E-09	9.011E-09	-	-1.285E-03	-	9.014E-09	9.013E-09	-	-9.933E-03	-
4.000E+03	6.796E-09	6.796E-09	-	-1.803E-04	-	6.757E-09	6.757E-09	-	-3.230E-03	-	6.759E-09	6.758E-09	-	-8.228E-03	-
5.000E+03	5.437E-09	5.436E-09	-	-1.057E-02	-	5.405E-09	5.405E-09	-	1.167E-03	-	5.406E-09	5.406E-09	-	7.227E-03	-
6.000E+03	4.530E-09	4.530E-09	-	2.266E-03	-	4.504E-09	4.504E-09	-	-6.432E-04	-	4.505E-09	4.505E-09	-	1.710E-03	-
8.000E+03	3.398E-09	3.397E-09	-	-2.257E-02	-	3.378E-09	3.378E-09	-	-1.078E-02	-	3.379E-09	3.378E-09	-	-1.580E-02	-
1.000E+04	2.718E-09	2.718E-09	-	-1.132E-02	-	2.702E-09	2.702E-09	-	6.117E-04	-	2.703E-09	2.703E-09	-	-1.187E-02	-
1.500E+04	1.812E-09	1.812E-09	-	8.549E-04	-	1.802E-09	1.802E-09	-	-2.429E-02	-	1.802E-09	1.802E-09	-	2.627E-04	-
2.000E+04	1.359E-09	1.359E-09	-	-3.819E-03	-	1.351E-09	1.351E-09	-	8.047E-03	-	1.351E-09	1.351E-09	-	3.259E-02	-
3.000E+04	9.060E-10	9.058E-10	-	-2.181E-02	-	9.007E-10	9.006E-10	-	-1.362E-02	-	9.010E-10	9.008E-10	-	-2.229E-02	-
4.000E+04	6.795E-10	6.796E-10	-	1.057E-02	-	6.756E-10	6.757E-10	-	7.602E-03	-	6.758E-10	6.758E-10	-	2.595E-03	-
5.000E+04	5.436E-10	5.436E-10	-	-8.822E-03	-	5.404E-10	5.404E-10	-	3.039E-03	-	5.406E-10	5.405E-10	-	-9.394E-03	-
6.000E+04	4.530E-10	4.529E-10	-	-1.157E-02	-	4.504E-10	4.503E-10	-	-1.453E-02	-	4.505E-10	4.504E-10	-	-1.219E-02	-
8.000E+04	3.397E-10	3.397E-10	-	2.439E-03	-	3.378E-10	3.377E-10	-	-1.520E-02	-	3.379E-10	3.378E-10	-	-2.022E-02	-
1.000E+05	2.718E-10	2.718E-10	-	-5.380E-04	-	2.702E-10	2.702E-10	-	1.125E-02	-	2.703E-10	2.703E-10	-	-1.156E-03	-

a = XCOM, b = Py-AMA.seidy and c = Py-MLBUF

Table S2: ϵ of MAC and LAC.

Photon Energy	MAC				LAC			
MeV	ϵ(B-C, Si-C)	ϵ(B-C, Al-C)	ϵ(B-C, d1/d3)	ϵ(B-C, d2/d4)	ϵ(B-C, Si-C)	ϵ(B-C, Al-C)	ϵ(B-C, d1/d3)	ϵ(B-C, d2/d4)
1.000E-03	-224.27E-03	-44.95E-03	158.25E-03	-31.52E-03	-1.28E+00	4.98E+00	-4.12E+00	2.47E+00
1.500E-03	-302.66E-03	0.00E+00	159.52E-03	-19.26E-03	-1.32E+00	5.00E+00	-4.13E+00	2.48E+00
2.000E-03	493.10E-03	-98.14E-03	-132.50E-03	-26.84E-03	-609.96E-03	4.97E+00	-4.44E+00	2.48E+00
3.000E-03	410.40E-03	-81.74E-03	-116.53E-03	-30.14E-03	-680.36E-03	4.96E+00	-4.40E+00	2.47E+00
4.000E-03	191.02E-03	-47.66E-03	-69.88E-03	-9.75E-03	-876.80E-03	5.01E+00	-4.35E+00	2.49E+00
5.000E-03	-394.74E-03	-33.03E-03	231.39E-03	-31.01E-03	-1.45E+00	4.98E+00	-4.05E+00	2.47E+00
6.000E-03	-428.49E-03	-53.79E-03	259.88E-03	-31.55E-03	-1.46E+00	4.98E+00	-4.04E+00	2.47E+00
8.000E-03	-567.64E-03	95.15E-03	209.91E-03	-3.10E-03	-1.55E+00	5.04E+00	-4.05E+00	2.50E+00
1.000E-02	-506.37E-03	17.55E-03	240.61E-03	-2.35E-03	-1.56E+00	5.04E+00	-4.05E+00	2.50E+00
1.500E-02	-544.37E-03	54.73E-03	222.11E-03	-1.12E-03	-1.57E+00	5.05E+00	-4.04E+00	2.50E+00
2.000E-02	-505.36E-03	12.39E-03	241.58E-03	-298.62E-06	-1.57E+00	5.05E+00	-4.05E+00	2.50E+00
2.634E-02	-510.34E-03	27.00E-03	226.06E-03	239.77E-06	-1.56E+00	5.05E+00	-4.05E+00	2.50E+00
3.000E-02	-501.54E-03	38.77E-03	214.61E-03	353.19E-06	-1.56E+00	5.05E+00	-4.05E+00	2.50E+00
4.000E-02	-499.17E-03	0.00E+00	243.68E-03	282.21E-06	-1.52E+00	5.05E+00	-4.07E+00	2.50E+00
5.000E-02	-401.38E-03	14.39E-03	165.59E-03	-310.49E-06	-1.47E+00	5.05E+00	-4.09E+00	2.50E+00
5.954E-02	-355.13E-03	0.00E+00	171.15E-03	-1.06E-03	-1.41E+00	5.04E+00	-4.12E+00	2.50E+00
6.000E-02	-339.49E-03	0.00E+00	170.99E-03	-1.09E-03	-1.41E+00	5.04E+00	-4.12E+00	2.50E+00
8.000E-02	-244.67E-03	0.00E+00	133.15E-03	-2.81E-03	-1.31E+00	5.04E+00	-4.17E+00	2.50E+00
1.000E-01	-139.15E-03	-46.45E-03	97.11E-03	-4.15E-03	-1.23E+00	5.03E+00	-4.20E+00	2.50E+00
1.500E-01	-65.02E-03	0.00E+00	70.37E-03	-5.96E-03	-1.13E+00	5.03E+00	-4.24E+00	2.50E+00
2.000E-01	-76.34E-03	0.00E+00	63.98E-03	-6.58E-03	-1.10E+00	5.02E+00	-4.25E+00	2.50E+00
3.000E-01	0.00E+00	0.00E+00	6.55E-03	-7.13E-03	-1.08E+00	5.02E+00	-4.26E+00	2.50E+00
4.000E-01	-10.40E-03	-10.41E-03	15.68E-03	-7.18E-03	-1.07E+00	5.02E+00	-4.27E+00	2.50E+00
5.000E-01	0.00E+00	-11.46E-03	9.32E-03	-7.26E-03	-1.07E+00	5.02E+00	-4.27E+00	2.50E+00
6.000E-01	0.00E+00	-12.43E-03	12.91E-03	-7.25E-03	-1.06E+00	5.02E+00	-4.27E+00	2.50E+00
6.620E-01	13.00E-03	-13.00E-03	10.15E-03	-7.26E-03	-1.06E+00	5.02E+00	-4.27E+00	2.50E+00
8.000E-01	0.00E+00	-14.20E-03	14.36E-03	-7.32E-03	-1.06E+00	5.02E+00	-4.27E+00	2.50E+00
1.000E+00	0.00E+00	-15.81E-03	17.27E-03	-7.32E-03	-1.06E+00	5.02E+00	-4.27E+00	2.50E+00
1.173E+00	17.13E-03	-17.13E-03	4.95E-03	-7.34E-03	-1.06E+00	5.02E+00	-4.27E+00	2.50E+00
1.333E+00	18.28E-03	-18.28E-03	8.84E-03	-7.32E-03	-1.06E+00	5.02E+00	-4.27E+00	2.50E+00
1.500E+00	0.00E+00	-19.41E-03	19.45E-03	-7.36E-03	-1.06E+00	5.02E+00	-4.27E+00	2.50E+00
2.000E+00	0.00E+00	-22.44E-03	18.00E-03	-7.34E-03	-1.06E+00	5.02E+00	-4.27E+00	2.50E+00
2.506E+00	0.00E+00	-25.06E-03	13.99E-03	-7.13E-03	-1.07E+00	5.02E+00	-4.27E+00	2.50E+00
3.000E+00	0.00E+00	0.00E+00	5.19E-03	-7.15E-03	-1.07E+00	5.02E+00	-4.26E+00	2.50E+00
4.000E+00	-30.84E-03	0.00E+00	5.49E-03	-7.19E-03	-1.08E+00	5.02E+00	-4.26E+00	2.50E+00
5.000E+00	-33.57E-03	0.00E+00	26.96E-03	-6.99E-03	-1.09E+00	5.02E+00	-4.26E+00	2.50E+00
6.000E+00	-35.61E-03	-35.63E-03	48.17E-03	-6.96E-03	-1.10E+00	5.02E+00	-4.25E+00	2.50E+00
8.000E+00	-38.37E-03	0.00E+00	22.56E-03	-6.74E-03	-1.12E+00	5.02E+00	-4.24E+00	2.50E+00
1.000E+01	-79.87E-03	0.00E+00	28.67E-03	-6.68E-03	-1.14E+00	5.02E+00	-4.24E+00	2.50E+00
1.500E+01	-82.95E-03	0.00E+00	56.77E-03	-6.35E-03	-1.16E+00	5.03E+00	-4.22E+00	2.50E+00
1.600E+01	-83.09E-03	-41.58E-03	75.74E-03	-6.34E-03	-1.17E+00	5.03E+00	-4.22E+00	2.50E+00
1.800E+01	-124.74E-03	0.00E+00	70.67E-03	-6.27E-03	-1.17E+00	5.03E+00	-4.22E+00	2.50E+00
2.000E+01	-124.53E-03	0.00E+00	68.13E-03	-6.22E-03	-1.18E+00	5.03E+00	-4.22E+00	2.50E+00
2.200E+01	-124.07E-03	0.00E+00	55.46E-03	-6.22E-03	-1.19E+00	5.03E+00	-4.21E+00	2.50E+00
2.400E+01	-123.51E-03	0.00E+00	85.90E-03	-6.18E-03	-1.19E+00	5.03E+00	-4.21E+00	2.50E+00
2.600E+01	-163.73E-03	0.00E+00	74.28E-03	-6.06E-03	-1.19E+00	5.03E+00	-4.21E+00	2.50E+00
2.800E+01	-162.73E-03	0.00E+00	83.56E-03	-6.01E-03	-1.20E+00	5.03E+00	-4.21E+00	2.50E+00
3.000E+01	-121.36E-03	0.00E+00	82.60E-03	-6.02E-03	-1.20E+00	5.03E+00	-4.21E+00	2.50E+00
4.000E+01	-156.80E-03	0.00E+00	80.98E-03	-5.93E-03	-1.21E+00	5.03E+00	-4.20E+00	2.50E+00
5.000E+01	-152.38E-03	0.00E+00	79.71E-03	-5.92E-03	-1.22E+00	5.03E+00	-4.20E+00	2.50E+00
6.000E+01	-148.64E-03	-37.22E-03	100.43E-03	-5.86E-03	-1.22E+00	5.03E+00	-4.20E+00	2.50E+00
8.000E+01	-178.51E-03	0.00E+00	98.02E-03	-5.76E-03	-1.23E+00	5.03E+00	-4.20E+00	2.50E+00
1.000E+02	-138.55E-03	-34.69E-03	97.80E-03	-5.68E-03	-1.23E+00	5.03E+00	-4.20E+00	2.50E+00
1.500E+02	-164.47E-03	0.00E+00	81.42E-03	-5.73E-03	-1.23E+00	5.03E+00	-4.19E+00	2.50E+00
2.000E+02	-191.08E-03	0.00E+00	84.34E-03	-5.63E-03	-1.23E+00	5.03E+00	-4.19E+00	2.50E+00
3.000E+02	-183.88E-03	0.00E+00	96.39E-03	-5.79E-03	-1.24E+00	5.03E+00	-4.19E+00	2.50E+00
4.000E+02	-179.75E-03	0.00E+00	105.23E-03	-5.81E-03	-1.24E+00	5.03E+00	-4.19E+00	2.50E+00
5.000E+02	-177.04E-03	0.00E+00	94.14E-03	-5.84E-03	-1.24E+00	5.03E+00	-4.19E+00	2.50E+00
6.000E+02	-145.94E-03	-29.23E-03	103.15E-03	-5.90E-03	-1.24E+00	5.03E+00	-4.19E+00	2.50E+00
8.000E+02	-172.56E-03	0.00E+00	81.29E-03	-5.97E-03	-1.24E+00	5.03E+00	-4.19E+00	2.50E+00
1.000E+03	-170.94E-03	0.00E+00	87.20E-03	-6.03E-03	-1.24E+00	5.03E+00	-4.19E+00	2.50E+00
1.500E+03	-168.54E-03	-28.14E-03	103.98E-03	-6.04E-03	-1.24E+00	5.03E+00	-4.19E+00	2.50E+00
2.000E+03	-167.27E-03	0.00E+00	78.99E-03	-5.97E-03	-1.24E+00	5.03E+00	-4.19E+00	2.50E+00
3.000E+03	-165.88E-03	0.00E+00	87.70E-03	-6.06E-03	-1.24E+00	5.03E+00	-4.19E+00	2.50E+00
4.000E+03	-165.11E-03	-27.56E-03	106.75E-03	-6.03E-03	-1.24E+00	5.03E+00	-4.19E+00	2.50E+00
5.000E+03	-164.65E-03	-27.49E-03	107.10E-03	-5.94E-03	-1.24E+00	5.03E+00	-4.19E+00	2.50E+00
6.000E+03	-164.34E-03	0.00E+00	85.08E-03	-6.06E-03	-1.24E+00	5.03E+00	-4.19E+00	2.50E+00
8.000E+03	36.61E-03	36.55E-03	36.54E-03	36.58E-03	-1.24E+00	5.03E+00	-4.19E+00	2.50E+00
1.000E+04	36.67E-03	36.61E-03	36.60E-03	36.64E-03	-1.24E+00	5.03E+00	-4.19E+00	2.50E+00
1.500E+04	36.76E-03	36.69E-03	36.69E-03	36.73E-03	-1.24E+00	5.03E+00	-4.19E+00	2.50E+00
2.000E+04	36.81E-03	36.74E-03	36.74E-03	36.77E-03	-1.24E+00	5.03E+00	-4.19E+00	2.50E+00
3.000E+04	36.85E-03	36.79E-03	36.79E-03	36.82E-03	-1.24E+00	5.03E+00	-4.19E+00	2.50E+00
4.000E+04	36.88E-03	36.81E-03	36.81E-03	36.85E-03	-1.24E+00	5.03E+00	-4.19E+00	2.50E+00
5.000E+04	36.89E-03	36.83E-03	36.83E-03	36.86E-03	-1.24E+00	5.03E+00	-4.19E+00	2.50E+00
6.000E+04	36.90E-03	36.84E-03	36.84E-03	36.87E-03	-1.24E+00	5.03E+00	-4.19E+00	2.50E+00
8.000E+04	36.92E-03	36.86E-03	36.86E-03	36.89E-03	-1.24E+00	5.03E+00	-4.19E+00	2.50E+00
1.000E+05	36.93E-03	36.86E-03	36.86E-03	36.90E-03	-1.24E+00	5.03E+00	-4.19E+00	2.50E+00

Table S3: ϵ of HVL and TVL.

Photon Energy	HVL				TVL			
MeV	ϵ(B-C, Si-C)	ϵ(B-C, Al-C)	ϵ(B-C, d1/d3)	ϵ(B-C, d2/d4)	ϵ(B-C, Si-C)	ϵ(B-C, Al-C)	ϵ(B-C, d1/d3)	ϵ(B-C, d2/d4)
1.000E-03	1.30E+00	-4.74E+00	4.30E+00	-2.41E+00	1.30E+00	-4.74E+00	4.30E+00	-2.41E+00
1.500E-03	1.34E+00	-4.76E+00	4.30E+00	-2.42E+00	1.34E+00	-4.76E+00	4.30E+00	-2.42E+00
2.000E-03	613.71E-03	-4.73E+00	4.65E+00	-2.42E+00	613.71E-03	-4.73E+00	4.65E+00	-2.42E+00
3.000E-03	685.02E-03	-4.73E+00	4.61E+00	-2.41E+00	685.02E-03	-4.73E+00	4.61E+00	-2.41E+00
4.000E-03	884.56E-03	-4.77E+00	4.55E+00	-2.43E+00	884.56E-03	-4.77E+00	4.55E+00	-2.43E+00
5.000E-03	1.47E+00	-4.75E+00	4.22E+00	-2.41E+00	1.47E+00	-4.75E+00	4.22E+00	-2.41E+00
6.000E-03	1.48E+00	-4.74E+00	4.21E+00	-2.41E+00	1.48E+00	-4.74E+00	4.21E+00	-2.41E+00
8.000E-03	1.57E+00	-4.80E+00	4.22E+00	-2.44E+00	1.57E+00	-4.80E+00	4.22E+00	-2.44E+00
1.000E-02	1.58E+00	-4.80E+00	4.22E+00	-2.44E+00	1.58E+00	-4.80E+00	4.22E+00	-2.44E+00
1.500E-02	1.60E+00	-4.80E+00	4.22E+00	-2.44E+00	1.60E+00	-4.80E+00	4.22E+00	-2.44E+00
2.000E-02	1.60E+00	-4.81E+00	4.22E+00	-2.44E+00	1.60E+00	-4.81E+00	4.22E+00	-2.44E+00
2.634E-02	1.59E+00	-4.81E+00	4.22E+00	-2.44E+00	1.59E+00	-4.81E+00	4.22E+00	-2.44E+00
3.000E-02	1.58E+00	-4.81E+00	4.23E+00	-2.44E+00	1.58E+00	-4.81E+00	4.23E+00	-2.44E+00
4.000E-02	1.54E+00	-4.81E+00	4.24E+00	-2.44E+00	1.54E+00	-4.81E+00	4.24E+00	-2.44E+00
5.000E-02	1.49E+00	-4.80E+00	4.27E+00	-2.44E+00	1.49E+00	-4.80E+00	4.27E+00	-2.44E+00
5.954E-02	1.43E+00	-4.80E+00	4.29E+00	-2.44E+00	1.43E+00	-4.80E+00	4.29E+00	-2.44E+00
6.000E-02	1.43E+00	-4.80E+00	4.30E+00	-2.44E+00	1.43E+00	-4.80E+00	4.30E+00	-2.44E+00
8.000E-02	1.32E+00	-4.79E+00	4.35E+00	-2.44E+00	1.32E+00	-4.79E+00	4.35E+00	-2.44E+00
1.000E-01	1.24E+00	-4.79E+00	4.38E+00	-2.44E+00	1.24E+00	-4.79E+00	4.38E+00	-2.44E+00
1.500E-01	1.15E+00	-4.78E+00	4.43E+00	-2.44E+00	1.15E+00	-4.78E+00	4.43E+00	-2.44E+00
2.000E-01	1.11E+00	-4.78E+00	4.44E+00	-2.44E+00	1.11E+00	-4.78E+00	4.44E+00	-2.44E+00
3.000E-01	1.09E+00	-4.78E+00	4.45E+00	-2.44E+00	1.09E+00	-4.78E+00	4.45E+00	-2.44E+00
4.000E-01	1.08E+00	-4.78E+00	4.46E+00	-2.44E+00	1.08E+00	-4.78E+00	4.46E+00	-2.44E+00
5.000E-01	1.08E+00	-4.78E+00	4.46E+00	-2.44E+00	1.08E+00	-4.78E+00	4.46E+00	-2.44E+00
6.000E-01	1.08E+00	-4.78E+00	4.46E+00	-2.44E+00	1.08E+00	-4.78E+00	4.46E+00	-2.44E+00
6.620E-01	1.07E+00	-4.78E+00	4.46E+00	-2.44E+00	1.07E+00	-4.78E+00	4.46E+00	-2.44E+00
8.000E-01	1.07E+00	-4.78E+00	4.46E+00	-2.44E+00	1.07E+00	-4.78E+00	4.46E+00	-2.44E+00
1.000E+00	1.07E+00	-4.78E+00	4.46E+00	-2.44E+00	1.07E+00	-4.78E+00	4.46E+00	-2.44E+00
1.173E+00	1.07E+00	-4.78E+00	4.46E+00	-2.44E+00	1.07E+00	-4.78E+00	4.46E+00	-2.44E+00
1.333E+00	1.07E+00	-4.78E+00	4.46E+00	-2.44E+00	1.07E+00	-4.78E+00	4.46E+00	-2.44E+00
1.500E+00	1.07E+00	-4.78E+00	4.46E+00	-2.44E+00	1.07E+00	-4.78E+00	4.46E+00	-2.44E+00
2.000E+00	1.08E+00	-4.78E+00	4.46E+00	-2.44E+00	1.08E+00	-4.78E+00	4.46E+00	-2.44E+00
2.506E+00	1.08E+00	-4.78E+00	4.46E+00	-2.44E+00	1.08E+00	-4.78E+00	4.46E+00	-2.44E+00
3.000E+00	1.09E+00	-4.78E+00	4.45E+00	-2.44E+00	1.09E+00	-4.78E+00	4.45E+00	-2.44E+00
4.000E+00	1.10E+00	-4.78E+00	4.45E+00	-2.44E+00	1.10E+00	-4.78E+00	4.45E+00	-2.44E+00
5.000E+00	1.11E+00	-4.78E+00	4.44E+00	-2.44E+00	1.11E+00	-4.78E+00	4.44E+00	-2.44E+00
6.000E+00	1.12E+00	-4.78E+00	4.44E+00	-2.44E+00	1.12E+00	-4.78E+00	4.44E+00	-2.44E+00
8.000E+00	1.14E+00	-4.78E+00	4.43E+00	-2.44E+00	1.14E+00	-4.78E+00	4.43E+00	-2.44E+00
1.000E+01	1.15E+00	-4.78E+00	4.42E+00	-2.44E+00	1.15E+00	-4.78E+00	4.42E+00	-2.44E+00
1.500E+01	1.18E+00	-4.78E+00	4.41E+00	-2.44E+00	1.18E+00	-4.78E+00	4.41E+00	-2.44E+00
1.600E+01	1.18E+00	-4.78E+00	4.41E+00	-2.44E+00	1.18E+00	-4.78E+00	4.41E+00	-2.44E+00
1.800E+01	1.19E+00	-4.79E+00	4.41E+00	-2.44E+00	1.19E+00	-4.79E+00	4.41E+00	-2.44E+00
2.000E+01	1.19E+00	-4.79E+00	4.40E+00	-2.44E+00	1.19E+00	-4.79E+00	4.40E+00	-2.44E+00
2.200E+01	1.20E+00	-4.79E+00	4.40E+00	-2.44E+00	1.20E+00	-4.79E+00	4.40E+00	-2.44E+00
2.400E+01	1.20E+00	-4.79E+00	4.40E+00	-2.44E+00	1.20E+00	-4.79E+00	4.40E+00	-2.44E+00
2.600E+01	1.21E+00	-4.79E+00	4.40E+00	-2.44E+00	1.21E+00	-4.79E+00	4.40E+00	-2.44E+00
2.800E+01	1.21E+00	-4.79E+00	4.39E+00	-2.44E+00	1.21E+00	-4.79E+00	4.39E+00	-2.44E+00
3.000E+01	1.21E+00	-4.79E+00	4.39E+00	-2.44E+00	1.21E+00	-4.79E+00	4.39E+00	-2.44E+00
4.000E+01	1.23E+00	-4.79E+00	4.39E+00	-2.44E+00	1.23E+00	-4.79E+00	4.39E+00	-2.44E+00
5.000E+01	1.23E+00	-4.79E+00	4.38E+00	-2.44E+00	1.23E+00	-4.79E+00	4.38E+00	-2.44E+00
6.000E+01	1.24E+00	-4.79E+00	4.38E+00	-2.44E+00	1.24E+00	-4.79E+00	4.38E+00	-2.44E+00
8.000E+01	1.24E+00	-4.79E+00	4.38E+00	-2.44E+00	1.24E+00	-4.79E+00	4.38E+00	-2.44E+00
1.000E+02	1.24E+00	-4.79E+00	4.38E+00	-2.44E+00	1.24E+00	-4.79E+00	4.38E+00	-2.44E+00
1.500E+02	1.25E+00	-4.79E+00	4.38E+00	-2.44E+00	1.25E+00	-4.79E+00	4.38E+00	-2.44E+00
2.000E+02	1.25E+00	-4.79E+00	4.38E+00	-2.44E+00	1.25E+00	-4.79E+00	4.38E+00	-2.44E+00
3.000E+02	1.25E+00	-4.79E+00	4.38E+00	-2.44E+00	1.25E+00	-4.79E+00	4.38E+00	-2.44E+00
4.000E+02	1.25E+00	-4.79E+00	4.37E+00	-2.44E+00	1.25E+00	-4.79E+00	4.37E+00	-2.44E+00
5.000E+02	1.25E+00	-4.79E+00	4.37E+00	-2.44E+00	1.25E+00	-4.79E+00	4.37E+00	-2.44E+00
6.000E+02	1.25E+00	-4.79E+00	4.37E+00	-2.44E+00	1.25E+00	-4.79E+00	4.37E+00	-2.44E+00
8.000E+02	1.25E+00	-4.79E+00	4.37E+00	-2.44E+00	1.25E+00	-4.79E+00	4.37E+00	-2.44E+00
1.000E+03	1.25E+00	-4.79E+00	4.37E+00	-2.44E+00	1.25E+00	-4.79E+00	4.37E+00	-2.44E+00
1.500E+03	1.25E+00	-4.79E+00	4.37E+00	-2.44E+00	1.25E+00	-4.79E+00	4.37E+00	-2.44E+00
2.000E+03	1.25E+00	-4.79E+00	4.37E+00	-2.44E+00	1.25E+00	-4.79E+00	4.37E+00	-2.44E+00
3.000E+03	1.25E+00	-4.79E+00	4.37E+00	-2.44E+00	1.25E+00	-4.79E+00	4.37E+00	-2.44E+00
4.000E+03	1.25E+00	-4.79E+00	4.37E+00	-2.44E+00	1.25E+00	-4.79E+00	4.37E+00	-2.44E+00
5.000E+03	1.25E+00	-4.79E+00	4.37E+00	-2.44E+00	1.25E+00	-4.79E+00	4.37E+00	-2.44E+00
6.000E+03	1.25E+00	-4.79E+00	4.37E+00	-2.44E+00	1.25E+00	-4.79E+00	4.37E+00	-2.44E+00
8.000E+03	1.25E+00	-4.79E+00	4.37E+00	-2.44E+00	1.25E+00	-4.79E+00	4.37E+00	-2.44E+00
1.000E+04	1.25E+00	-4.79E+00	4.37E+00	-2.44E+00	1.25E+00	-4.79E+00	4.37E+00	-2.44E+00
1.500E+04	1.25E+00	-4.79E+00	4.37E+00	-2.44E+00	1.25E+00	-4.79E+00	4.37E+00	-2.44E+00
2.000E+04	1.25E+00	-4.79E+00	4.37E+00	-2.44E+00	1.25E+00	-4.79E+00	4.37E+00	-2.44E+00
3.000E+04	1.25E+00	-4.79E+00	4.37E+00	-2.44E+00	1.25E+00	-4.79E+00	4.37E+00	-2.44E+00
4.000E+04	1.25E+00	-4.79E+00	4.37E+00	-2.44E+00	1.25E+00	-4.79E+00	4.37E+00	-2.44E+00
5.000E+04	1.25E+00	-4.79E+00	4.37E+00	-2.44E+00	1.25E+00	-4.79E+00	4.37E+00	-2.44E+00
6.000E+04	1.25E+00	-4.79E+00	4.37E+00	-2.44E+00	1.25E+00	-4.79E+00	4.37E+00	-2.44E+00
8.000E+04	1.25E+00	-4.79E+00	4.37E+00	-2.44E+00	1.25E+00	-4.79E+00	4.37E+00	-2.44E+00
1.000E+05	1.25E+00	-4.79E+00	4.37E+00	-2.44E+00	1.25E+00	-4.79E+00	4.37E+00	-2.44E+00

Table S4: ϵ of σ-atomic, σ-electron, photoelectric absorption and Z_{eff} .

Photon Energy	σ-atomic		σ-electron		photoelectric absorption		Z_{eff}	
MeV	ϵ(B-C, Si-C)	ϵ(B-C, Al-C)	ϵ(B-C, Si-C)	ϵ(B-C, Al-C)	ϵ(B-C, Si-C)	ϵ(B-C, Al-C)	ϵ(B-C, Si-C)	ϵ(B-C, Al-C)
1.000E-03	-400.44E-03	-25.54E-03	-153.02E-03	-70.56E-03	-224.42E-03	-44.98E-03	-247.80E-03	45.05E-03
1.500E-03	-441.82E-03	-435.50E-06	-212.48E-03	-40.28E-03	-242.57E-03	-60.79E-03	-229.83E-03	39.86E-03
2.000E-03	277.10E-03	-32.49E-03	405.07E-03	-5.15E-03	494.07E-03	-98.33E-03	-127.46E-03	-27.34E-03
3.000E-03	206.08E-03	-37.27E-03	344.72E-03	-9.07E-03	412.54E-03	-82.17E-03	-138.16E-03	-28.20E-03
4.000E-03	7.88E-03	7.42E-03	176.38E-03	18.36E-03	192.49E-03	-48.03E-03	-168.21E-03	-10.93E-03
5.000E-03	-568.81E-03	-20.49E-03	-423.97E-03	-14.66E-03	-363.52E-03	-66.33E-03	-145.45E-03	-5.83E-03
6.000E-03	-578.54E-03	-21.31E-03	-437.09E-03	-15.90E-03	-377.36E-03	-54.11E-03	-142.07E-03	-5.41E-03
8.000E-03	-669.72E-03	36.86E-03	-531.53E-03	25.19E-03	-477.55E-03	0.00E+00	-138.92E-03	11.66E-03
1.000E-02	-679.58E-03	38.58E-03	-544.75E-03	26.11E-03	-495.40E-03	0.00E+00	-135.58E-03	12.47E-03
1.500E-02	-691.62E-03	41.30E-03	-559.85E-03	27.57E-03	-560.22E-03	56.34E-03	-132.51E-03	13.73E-03
2.000E-02	-693.77E-03	42.96E-03	-559.99E-03	28.37E-03	-531.78E-03	13.04E-03	-134.54E-03	14.58E-03
2.634E-02	-686.10E-03	43.83E-03	-544.01E-03	28.23E-03	-558.50E-03	29.56E-03	-142.87E-03	15.60E-03
3.000E-02	-677.33E-03	43.84E-03	-528.18E-03	27.65E-03	-521.74E-03	0.00E+00	-149.93E-03	16.18E-03
4.000E-02	-639.88E-03	42.78E-03	-467.10E-03	25.16E-03	-552.08E-03	20.95E-03	-173.59E-03	17.62E-03
5.000E-02	-588.27E-03	40.36E-03	-392.65E-03	21.62E-03	-537.86E-03	20.80E-03	-196.39E-03	18.74E-03
5.954E-02	-533.82E-03	37.57E-03	-323.09E-03	18.33E-03	-568.18E-03	35.71E-03	-211.42E-03	19.24E-03
6.000E-02	-531.24E-03	37.45E-03	-319.98E-03	18.19E-03	-545.65E-03	36.58E-03	-211.95E-03	19.25E-03
8.000E-02	-424.77E-03	31.47E-03	-204.98E-03	12.55E-03	-535.24E-03	0.00E+00	-220.24E-03	18.92E-03
1.000E-01	-346.92E-03	26.96E-03	-134.13E-03	9.15E-03	-556.15E-03	18.04E-03	-213.08E-03	17.81E-03
1.500E-01	-250.07E-03	21.06E-03	-57.87E-03	5.35E-03	-574.71E-03	64.23E-03	-192.31E-03	15.71E-03
2.000E-01	-215.19E-03	19.01E-03	-33.11E-03	4.21E-03	-576.41E-03	31.34E-03	-182.14E-03	14.79E-03
3.000E-01	-192.32E-03	17.37E-03	-17.51E-03	3.22E-03	-582.32E-03	53.25E-03	-174.84E-03	14.15E-03
4.000E-01	-185.47E-03	17.11E-03	-12.99E-03	3.14E-03	-579.92E-03	24.30E-03	-172.50E-03	13.97E-03
5.000E-01	-182.50E-03	16.88E-03	-11.03E-03	2.99E-03	-574.97E-03	22.24E-03	-171.50E-03	13.89E-03
6.000E-01	-180.99E-03	16.87E-03	-10.03E-03	3.01E-03	-563.78E-03	35.44E-03	-170.98E-03	13.86E-03
6.620E-01	-180.39E-03	16.83E-03	-9.64E-03	2.99E-03	-584.27E-03	0.00E+00	-170.77E-03	13.84E-03
8.000E-01	-179.44E-03	16.70E-03	-9.01E-03	2.88E-03	-554.79E-03	0.00E+00	-170.45E-03	13.82E-03
1.000E+00	-178.70E-03	16.67E-03	-8.52E-03	2.86E-03	-590.99E-03	33.65E-03	-170.20E-03	13.81E-03
1.173E+00	-178.44E-03	16.62E-03	-8.35E-03	2.83E-03	-591.99E-03	31.34E-03	-170.11E-03	13.79E-03
1.333E+00	-178.53E-03	16.67E-03	-8.40E-03	2.88E-03	-584.57E-03	19.60E-03	-170.14E-03	13.79E-03
1.500E+00	-178.95E-03	16.61E-03	-8.68E-03	2.83E-03	-601.97E-03	48.45E-03	-170.29E-03	13.78E-03
2.000E+00	-181.57E-03	16.69E-03	-10.32E-03	2.93E-03	-583.20E-03	0.00E+00	-171.26E-03	13.77E-03
2.506E+00	-186.14E-03	17.23E-03	-13.33E-03	3.50E-03	-603.73E-03	55.22E-03	-172.83E-03	13.73E-03
3.000E+00	-190.89E-03	17.30E-03	-16.42E-03	3.57E-03	-569.40E-03	71.58E-03	-174.50E-03	13.73E-03
4.000E+00	-201.34E-03	17.46E-03	-23.29E-03	3.85E-03	-578.58E-03	21.16E-03	-178.09E-03	13.61E-03
5.000E+00	-211.95E-03	18.11E-03	-30.48E-03	4.59E-03	-574.31E-03	14.09E-03	-181.52E-03	13.53E-03
6.000E+00	-222.17E-03	18.42E-03	-37.54E-03	5.03E-03	-579.05E-03	35.30E-03	-184.70E-03	13.39E-03
8.000E+00	-240.04E-03	19.29E-03	-50.25E-03	6.11E-03	-593.03E-03	24.86E-03	-189.88E-03	13.18E-03
1.000E+01	-254.88E-03	19.76E-03	-61.18E-03	6.81E-03	-575.08E-03	32.13E-03	-193.81E-03	12.94E-03
1.500E+01	-281.60E-03	21.06E-03	-81.86E-03	8.60E-03	-601.81E-03	50.45E-03	-199.90E-03	12.46E-03
1.600E+01	-285.51E-03	21.17E-03	-84.98E-03	8.79E-03	-538.21E-03	0.00E+00	-200.71E-03	12.38E-03
1.800E+01	-292.71E-03	21.49E-03	-90.82E-03	9.27E-03	-611.25E-03	61.50E-03	-202.07E-03	12.22E-03
2.000E+01	-298.68E-03	21.72E-03	-95.74E-03	9.63E-03	-616.02E-03	68.87E-03	-203.14E-03	12.09E-03
2.200E+01	-303.68E-03	21.84E-03	-99.88E-03	9.85E-03	-606.06E-03	0.00E+00	-204.00E-03	11.98E-03
2.400E+01	-308.15E-03	22.04E-03	-103.67E-03	10.16E-03	-581.88E-03	83.61E-03	-204.69E-03	11.88E-03
2.600E+01	-312.07E-03	22.35E-03	-107.01E-03	10.58E-03	-542.99E-03	0.00E+00	-205.27E-03	11.77E-03
2.800E+01	-315.61E-03	22.54E-03	-110.07E-03	10.86E-03	-586.51E-03	0.00E+00	-205.76E-03	11.68E-03
3.000E+01	-318.46E-03	22.59E-03	-112.53E-03	10.98E-03	-578.34E-03	31.73E-03	-206.16E-03	11.61E-03
4.000E+01	-329.10E-03	23.03E-03	-121.94E-03	11.76E-03	-581.73E-03	28.54E-03	-207.41E-03	11.27E-03
5.000E+01	-335.59E-03	23.21E-03	-127.78E-03	12.13E-03	-571.63E-03	17.97E-03	-208.07E-03	11.08E-03
6.000E+01	-339.79E-03	23.42E-03	-131.63E-03	12.48E-03	-581.65E-03	21.67E-03	-208.44E-03	10.93E-03
8.000E+01	-344.99E-03	23.75E-03	-136.43E-03	12.98E-03	-577.87E-03	29.06E-03	-208.84E-03	10.76E-03
1.000E+02	-348.04E-03	23.98E-03	-139.28E-03	13.29E-03	-580.13E-03	36.47E-03	-209.05E-03	10.68E-03
1.500E+02	-351.64E-03	23.96E-03	-142.63E-03	13.35E-03	-601.09E-03	54.98E-03	-209.31E-03	10.62E-03
2.000E+02	-353.31E-03	24.20E-03	-144.20E-03	13.62E-03	-584.37E-03	0.00E+00	-209.41E-03	10.58E-03
3.000E+02	-354.53E-03	23.92E-03	-145.33E-03	13.33E-03	-582.16E-03	33.15E-03	-209.50E-03	10.59E-03
4.000E+02	-355.06E-03	23.89E-03	-145.82E-03	13.30E-03	-586.51E-03	29.50E-03	-209.54E-03	10.59E-03
5.000E+02	-355.30E-03	23.84E-03	-146.05E-03	13.25E-03	-568.60E-03	18.45E-03	-209.56E-03	10.58E-03
6.000E+02	-355.54E-03	23.73E-03	-146.27E-03	13.14E-03	-572.69E-03	22.15E-03	-209.58E-03	10.60E-03
8.000E+02	-355.61E-03	23.59E-03	-146.34E-03	13.00E-03	-558.33E-03	0.00E+00	-209.58E-03	10.59E-03
1.000E+03	-355.60E-03	23.47E-03	-146.32E-03	12.88E-03	-587.80E-03	36.95E-03	-209.58E-03	10.59E-03
1.500E+03	-355.73E-03	23.46E-03	-146.45E-03	12.87E-03	-606.39E-03	55.46E-03	-209.59E-03	10.59E-03
2.000E+03	-355.86E-03	23.59E-03	-146.59E-03	13.02E-03	-588.24E-03	0.00E+00	-209.58E-03	10.57E-03
3.000E+03	-355.78E-03	23.43E-03	-146.50E-03	12.84E-03	-584.73E-03	33.29E-03	-209.59E-03	10.59E-03
4.000E+03	-355.69E-03	23.47E-03	-146.43E-03	12.91E-03	-573.87E-03	29.60E-03	-209.57E-03	10.56E-03
5.000E+03	-355.85E-03	23.65E-03	-146.58E-03	13.09E-03	-588.56E-03	18.50E-03	-209.57E-03	10.56E-03
6.000E+03	-355.58E-03	23.42E-03	-146.32E-03	12.85E-03	-573.95E-03	22.20E-03	-209.57E-03	10.57E-03
8.000E+03	-355.79E-03	23.39E-03	-146.52E-03	12.83E-03	-588.58E-03	29.60E-03	-209.57E-03	10.57E-03
1.000E+04	-355.62E-03	23.46E-03	-146.37E-03	12.90E-03	-588.67E-03	37.01E-03	-209.56E-03	10.55E-03
1.500E+04	-355.80E-03	23.58E-03	-146.53E-03	13.02E-03	-551.88E-03	0.00E+00	-209.57E-03	10.56E-03
2.000E+04	-355.83E-03	23.62E-03	-146.57E-03	13.06E-03	-588.67E-03	0.00E+00	-209.57E-03	10.56E-03
3.000E+04	-355.57E-03	23.29E-03	-146.31E-03	12.71E-03	-584.99E-03	33.31E-03	-209.57E-03	10.58E-03
4.000E+04	-355.67E-03	23.30E-03	-146.41E-03	12.74E-03	-573.95E-03	29.60E-03	-209.56E-03	10.57E-03
5.000E+04	-355.77E-03	23.51E-03	-146.51E-03	12.95E-03	-588.67E-03	37.01E-03	-209.56E-03	10.56E-03
6.000E+04	-355.59E-03	23.23E-03	-146.33E-03	12.66E-03	-573.95E-03	22.20E-03	-209.57E-03	10.58E-03
8.000E+04	-355.77E-03	23.52E-03	-146.52E-03	12.96E-03	-559.32E-03	29.60E-03	-209.56E-03	10.56E-03
1.000E+05	-355.57E-03	23.34E-03	-146.31E-03	12.77E-03	-588.67E-03	37.01E-03	-209.57E-03	10.57E-03

Table S5: ϵ of EBF, EABF, DLEBF and DLEBF.

Photon Energy	EBF		EABF		DLEBF			DLEABF		
MeV	ϵ(B-C, Si-C)	ϵ(B-C, Al-C)	ϵ(B-C, Si-C)	ϵ(B-C, Al-C)	ϵ(d1, d2)	ϵ(d1, d3)	ϵ(d1, d4)	ϵ(d1, d2)	ϵ(d1, d3)	ϵ(d1, d4)
1.500E-02	-9.84E-03	-9.84E-03	-9.85E-03	-9.85E-03	0.00E+00	1.08E-01	1.08E-01	0.00E+00	9.80E-02	9.80E-02
2.000E-02	-9.63E-03	-9.63E-03	-1.92E-02	-1.92E-02	0.00E+00	2.76E-01	2.76E-01	0.00E+00	2.57E-01	2.57E-01
2.634E-02	2.15E+00	2.15E+00	2.48E+00	2.48E+00	0.00E+00	-2.27E+00	-2.27E+00	0.00E+00	-2.18E+00	-2.18E+00
3.000E-02	-4.62E-02	-4.62E-02	-3.70E-02	-2.77E-02	0.00E+00	5.85E-01	5.85E-01	0.00E+00	5.76E-01	5.76E-01
4.000E-02	-7.59E-02	-6.75E-02	-8.42E-02	-8.42E-02	7.94E-03	1.29E+00	1.29E+00	7.93E-03	1.31E+00	1.31E+00
5.000E-02	-1.05E-01	-1.05E-01	-1.11E-01	-1.04E-01	6.80E-03	2.13E+00	2.13E+00	1.33E-02	2.19E+00	2.20E+00
5.954E-02	-1.36E-01	-1.29E-01	-1.37E-01	-1.24E-01	1.17E-02	3.01E+00	3.01E+00	1.11E-02	3.23E+00	3.24E+00
6.000E-02	-1.48E-01	-1.42E-01	-1.36E-01	-1.29E-01	1.16E-02	3.05E+00	3.06E+00	1.10E-02	3.29E+00	3.29E+00
8.000E-02	-1.52E-01	-1.41E-01	-2.35E-01	-2.21E-01	1.32E-02	4.65E+00	4.65E+00	1.74E-02	5.03E+00	5.03E+00
1.000E-01	-2.08E-01	-1.98E-01	-2.13E-01	-1.98E-01	1.80E-02	5.86E+00	5.87E+00	1.97E-02	6.57E+00	6.57E+00
1.500E-01	-1.46E-01	-1.38E-01	-1.10E-01	-1.04E-01	1.41E-02	7.28E+00	7.28E+00	1.32E-02	8.42E+00	8.43E+00
2.000E-01	-8.35E-02	-7.94E-02	-3.03E-02	-3.03E-02	1.05E-02	7.83E+00	7.83E+00	7.81E-03	9.04E+00	9.04E+00
3.000E-01	-6.86E-02	-6.86E-02	3.17E-03	3.17E-03	5.23E-03	8.12E+00	8.12E+00	3.52E-03	9.22E+00	9.22E+00
4.000E-01	-3.11E-02	-2.66E-02	-1.41E-02	-1.06E-02	5.42E-03	8.09E+00	8.09E+00	3.98E-03	9.03E+00	9.03E+00
5.000E-01	-3.23E-02	-3.23E-02	7.66E-03	7.66E-03	5.70E-03	7.95E+00	7.96E+00	2.21E-03	8.76E+00	8.76E+00
6.000E-01	-3.32E-02	-3.32E-02	8.16E-03	8.16E-03	2.96E-03	7.79E+00	7.79E+00	2.40E-03	8.51E+00	8.51E+00
6.620E-01	1.93E-02	2.42E-02	0.00E+00	0.00E+00	0.00E+00	-7.13E+00	-7.13E+00	-2.31E-03	-7.71E+00	-7.71E+00
8.000E-01	-2.49E-02	-2.49E-02	-4.45E-03	-4.45E-03	6.38E-03	7.46E+00	7.46E+00	2.71E-03	8.05E+00	8.05E+00
1.000E+00	-1.55E-02	-1.04E-02	-9.44E-03	-9.44E-03	3.39E-03	7.14E+00	7.15E+00	2.97E-03	7.66E+00	7.66E+00
1.173E+00	1.06E-02	1.06E-02	4.88E-03	4.88E-03	0.00E+00	-6.47E+00	-6.48E+00	-2.92E-03	-6.87E+00	-6.87E+00
1.333E+00	1.62E-02	1.62E-02	0.00E+00	0.00E+00	-3.41E-03	-6.30E+00	-6.30E+00	-3.07E-03	-6.66E+00	-6.66E+00
1.500E+00	-1.64E-02	-1.64E-02	1.03E-02	1.03E-02	3.74E-03	6.56E+00	6.56E+00	0.00E+00	6.91E+00	6.91E+00
2.000E+00	5.70E-03	1.14E-02	5.45E-03	5.45E-03	0.00E+00	-5.77E+00	-5.77E+00	-3.54E-03	-6.00E+00	-6.00E+00
2.506E+00	5.88E-03	5.88E-03	5.70E-03	5.70E-03	0.00E+00	-5.48E+00	-5.48E+00	0.00E+00	-5.64E+00	-5.64E+00
3.000E+00	-6.02E-03	-6.02E-03	0.00E+00	0.00E+00	0.00E+00	5.53E+00	5.53E+00	4.30E-03	5.64E+00	5.64E+00
4.000E+00	-6.30E-03	-6.30E-03	-6.28E-03	-6.28E-03	6.10E-03	8.85E-01	8.85E-01	0.00E+00	5.07E+00	5.07E+00
5.000E+00	-7.92E-02	-6.60E-03	-6.34E-01	-1.98E-02	8.17E-02	4.72E+00	4.77E+00	5.47E-01	4.77E+00	5.20E+00
6.000E+00	6.83E-03	6.83E-03	0.00E+00	0.00E+00	0.00E+00	-4.20E+00	-4.20E+00	-5.30E-03	-4.05E+00	-4.05E+00
8.000E+00	7.23E-03	7.23E-03	7.43E-03	7.43E-03	0.00E+00	-3.72E+00	-3.72E+00	0.00E+00	-3.49E+00	-3.49E+00
1.000E+01	0.00E+00	0.00E+00	7.79E-03	7.79E-03	0.00E+00	-3.34E+00	-3.34E+00	0.00E+00	-3.06E+00	-3.06E+00
1.500E+01	-1.61E-02	-2.42E-02	1.69E-02	1.69E-02	0.00E+00	-2.63E+00	-2.64E+00	0.00E+00	-2.30E+00	-2.30E+00
